# PMCA-Based Detection of Prions in the Olfactory Mucosa of Patients With Sporadic Creutzfeldt–Jakob Disease

**DOI:** 10.3389/fnagi.2022.848991

**Published:** 2022-03-25

**Authors:** Federico Angelo Cazzaniga, Edoardo Bistaffa, Chiara Maria Giulia De Luca, Sara Maria Portaleone, Marcella Catania, Veronica Redaelli, Irene Tramacere, Giuseppe Bufano, Martina Rossi, Paola Caroppo, Anna Rita Giovagnoli, Pietro Tiraboschi, Giuseppe Di Fede, Roberto Eleopra, Grazia Devigili, Antonio Emanuele Elia, Roberto Cilia, Michele Fiorini, Matilde Bongianni, Giulia Salzano, Luigi Celauro, Federico Giuseppe Quarta, Angela Mammana, Giuseppe Legname, Fabrizio Tagliavini, Piero Parchi, Gianluigi Zanusso, Giorgio Giaccone, Fabio Moda

**Affiliations:** ^1^Unit of Neurology 5 and Neuropathology, Fondazione IRCCS Istituto Neurologico Carlo Besta, Milan, Italy; ^2^Department of Neuroscience, Scuola Internazionale Superiore di Studi Avanzati (SISSA), Trieste, Italy; ^3^Department of Health Sciences, Otolaryngology Unit, ASST Santi Paolo e Carlo Hospital, Università degli Studi di Milano, Milan, Italy; ^4^Department of Research and Clinical Development, Scientific Directorate, Fondazione IRCCS Istituto Neurologico Carlo Besta, Milan, Italy; ^5^Unit of Neurology 1 – Parkinson’s and Movement Disorders Unit, Fondazione IRCCS Istituto Neurologico Carlo Besta, Milan, Italy; ^6^Department of Neurosciences, Biomedicine and Movement Sciences, University of Verona, Verona, Italy; ^7^IRCCS, Istituto delle Scienze Neurologiche di Bologna (ISNB), Bologna, Italy; ^8^Scientific Directorate, Fondazione IRCCS Istituto Neurologico Carlo Besta, Milan, Italy; ^9^Department of Diagnostic Experimental and Specialty Medicine (DIMES), University of Bologna, Bologna, Italy

**Keywords:** Creutzfeldt–Jakob disease, olfactory mucosa, protein misfolding cyclic amplification, neurodegeneration, prion, peripheral biomarker

## Abstract

Sporadic Creutzfeldt-Jakob disease (sCJD) is a rare neurodegenerative disorder caused by the conformational conversion of the prion protein (PrP^C^) into an abnormally folded form, named prion (or PrP^Sc^). The combination of the polymorphism at codon 129 of the PrP gene (coding either methionine or valine) with the biochemical feature of the proteinase-K resistant PrP (generating either PrP^Sc^ type 1 or 2) gives rise to different PrP^Sc^ strains, which cause variable phenotypes of sCJD. The definitive diagnosis of sCJD and its classification can be achieved only post-mortem after PrP^Sc^ identification and characterization in the brain. By exploiting the Real-Time Quaking-Induced Conversion (RT-QuIC) assay, traces of PrP^Sc^ were found in the olfactory mucosa (OM) of sCJD patients, thus demonstrating that PrP^Sc^ is not confined to the brain. Here, we have optimized another technique, named protein misfolding cyclic amplification (PMCA) for detecting PrP^Sc^ in OM samples of sCJD patients. OM samples were collected from 27 sCJD and 2 genetic CJD patients (E200K). Samples from 34 patients with other neurodegenerative disorders were included as controls. Brains were collected from 26 sCJD patients and 16 of them underwent OM collection. Brain and OM samples were subjected to PMCA using the brains of transgenic mice expressing human PrP^C^ with methionine at codon 129 as reaction substrates. The amplified products were analyzed by Western blot after proteinase K digestion. Quantitative PMCA was performed to estimate PrP^Sc^ concentration in OM. PMCA enabled the detection of prions in OM samples with 79.3% sensitivity and 100% specificity. Except for a few cases, a predominant type 1 PrP^Sc^ was generated, regardless of the tissues analyzed. Notably, all amplified PrP^Sc^ were less resistant to PK compared to the original strain. In conclusion, although the optimized PMCA did not consent to recognize sCJD subtypes from the analysis of OM collected from living patients, it enabled us to estimate for the first time the amount of prions accumulating in this biological tissue. Further assay optimizations are needed to faithfully amplify peripheral prions whose recognition could lead to a better diagnosis and selection of patients for future clinical trials.

## Introduction

Human prion diseases are a group of rare and fatal neurodegenerative disorders with an incidence of 1.5–2 cases per million population/year, worldwide (Ladogana et al., 2005). They can have sporadic (85%) or genetic (5–15%) origins and can be acquired by infection through different routes (less than 5%) (Cochius et al., 1992; Bruce et al., 1997; Heckmann et al., 1997; Hill et al., 1997; Heath et al., 2006; Hamaguchi et al., 2009; Davidson et al., 2014). Sporadic forms are the most common, have unknown etiology, and include: Creutzfeldt-Jakob disease (sCJD) (Parchi et al., 1996), fatal insomnia (sFI), and variably protease-sensitive prionopathy (VPSPr) (Gambetti et al., 2008). The pathological agent responsible for these diseases is an abnormally folded protein, referred to as prion (or PrP^Sc^), which results from the conformational conversion of the physiological PrP^C^ (Prusiner, 1982). PrP^C^ is a glycophosphatidylinositol (GPI)-anchored glycoprotein encoded by the *PRNP* gene that has two common alleles encoding either methionine (M) or valine (V) at codon 129. These polymorphisms play an important role in modulating the clinicopathological phenotypes of prion diseases (Stahl et al., 1987; Tahiri-Alaoui et al., 2004). PrP^C^ possesses two *N*-glycosylation sites at asparagine 181 and 197 which allow the formation of di-glycosylated, mono-glycosylated, and un-glycosylated species. The relative abundance of each glycosylated species defines the glycoform ratio of both PrP^C^ and PrP^Sc^ (Rudd et al., 1999; Bate et al., 2016).

PrP^C^ is soluble in mild detergents and is completely degraded by proteolytic enzymes, including the proteinase K (PK) (Meyer et al., 1986). In contrast, PrP^Sc^ is partially insoluble in detergents and is more resistant to PK treatment, thus leaving a C-terminal and PK-resistant core (PrP^res^) after digestion (Pan et al., 1993). The digestion leads to the generation of amino-terminal-truncated fragments of di-glycosylated, mono-glycosylated or un-glycosylated PrP^res^ that migrate at lower molecular weights compared to those of PrP^C^ (Oesch et al., 1985). Of particular interest, the molecular weight of the un-glycosylated species can be either 21 or 19 kDa thus giving rise to type 1 or type 2 PrP^res^, respectively (Parchi et al., 2009). Thus, based on the un-glycosylated PrP^res^ fragment sizes (type 1 or 2) and the polymorphism at codon 129 of *PRNP* (M or V), sCJD can be classified in six different phenotypic subtypes: MM1, MM2, MV1, MV2, VV1, and VV2. Each sCJD subtype is characterized by specific clinical and neuropathological features (e.g., disease duration, clinical signs, neuropathological features, PrP^Sc^ tissue tropism) (Parchi et al., 1996, 1999, 2012; Puoti et al., 2012; Ironside et al., 2014; Ritchie and Ironside, 2017). The most frequent subtypes are MM1, VV2, and MV2 (Zerr and Parchi, 2018).

The presence of methionine or valine at codon 129 of PrP^C^, as well as other probably unknown factors, influences the structural rearrangement of the protein during misfolding and gives rise to different PrP^Sc^ strains which ultimately lead to the phenotypic heterogeneity of sCJD (Hosszu et al., 2004; Safar, 2012; Kobayashi et al., 2013, 2015). In some cases, sCJD might present with mixed phenotypic features characterized by a combination of type 1 and type 2 PrP^res^ (MM1 + 2, VV1 + 2 and MV1 + 2), which make the classification of the disease very challenging (Puoti et al., 1999; Uro-Coste et al., 2008; Parchi et al., 2009; Cassard et al., 2020). Moreover, several studies showed that prions can undergo processes of selection and adaptation. These phenomena can be sustained by two different hypotheses: (1) the cloud hypothesis, where the prion strain is intrinsically composed of a heterogeneous pool of PrP^Sc^ and only the variant able to replicate in the environment receives a selective advantage (Collinge, 2010; Li et al., 2010; Morales et al., 2016), and the (2) deformed templating hypothesis, where the prion strain is considered to be pure and can generate different PrP^Sc^ conformers starting from PrP^C^. In this case, the newly formed PrP^Sc^ variant that fits well to the environment will emerge (Makarava and Baskakov, 2012, 2013; Baskakov, 2014). These mechanisms may explain the resistance of prions to therapeutic treatments and suggest that drug-resistant PrP^Sc^ variants might emerge in patients under pharmacological treatment (Kabir and Safar, 2014). Once formed, PrP^Sc^ can interact with PrP^C^ acting as a template for its further conversion into PrP^Sc^ (Caughey, 2001). By exploiting this mechanism, PrP^Sc^ molecules spread throughout the central nervous system (CNS) and sustain disease progression (Caughey, 2001). At present, PrP^Sc^ is the only reliable biomarker of prion diseases and accumulates at high levels in the CNS. Thus, the definite diagnosis of sCJD requires PrP^Sc^ detection and characterization in the CNS through biopsy or postmortem [Federspil et al., 2002; Kübler et al., 2003; World Health Organisation [WHO], 2003]. This will allow us to define the PrP^res^ type (1 or 2) and categorize sCJD patients into one of the six subtypes. *Premortem* diagnosis relies on criteria which combine clinical (e.g., neuropsychiatric symptoms), instrumental (e.g., brain MRI and EEG abnormalities) and laboratory tests (variations in the CSF of 14.3.3 or tau protein, and RT-QuIC) that classify the disease as probable or possible (Hermann et al., 2021).

The RT-QuIC analysis has been recently developed and uses recombinant PrP (rec-PrP) as a reaction substrate and demonstrated the presence of PrP^Sc^ in the CSF, olfactory mucosa (OM), and skin of sCJD patients (Orrú et al., 2014, 2017; Franceschini et al., 2017; Mammana et al., 2020). Considering its reliability and robustness, the RT-QuIC has been introduced in several countries, including Italy and United States, among the clinical diagnostic criteria for human prion diseases (Istituto Superiore di Sanità; Watson et al., 2022). Unfortunately, this technique does not provide any information on PrP typing and the final reaction products seem to be not infectious (Raymond et al., 2020). In contrast, the PMCA uses PrP^C^ proteins either derived from brain homogenates or cell lysates and showed the presence of PrP^Sc^ in the CSF (Barria et al., 2018), urine (Moda et al., 2014), and blood (Concha-Marambio et al., 2016) of patients with variant CJD (vCJD), which is related to the consumption of foodstuff obtained from cattle affected by bovine spongiform encephalopathy (Hill et al., 1997). Notably, the PMCA generated products retained the biochemical and infectious properties of the original vCJD strain (Cali et al., 2019). Thus, in contrast to RT-QuIC, the PMCA might faithfully amplify PrP^Sc^ and offers the possibility to detect and recognize its type starting from the analysis of peripheral tissues of living patients.

Besides the vCJD strain, the prion strains responsible for sCJD have been barely amplified with PMCA (Jones et al., 2009; Moda et al., 2014; Concha-Marambio et al., 2016; Camacho et al., 2019). More recently, Bélondrade et al. (2021) have further optimized the PMCA to amplify sCJD using different reaction substrates and found that the amplification efficiency was seed- and substrate-dependent.

In our study, we describe a further optimization of the PMCA protocol that enables amplification of all sCJD prions present in the brain but also in the OM collected from 129MM, 129MV, and 129VV patients. Through quantitative PMCA, we could estimate the PrP^Sc^ concentration in the OM and verify whether and to what extent the prions amplified from both tissues maintained the original strain features, especially in terms of PK resistance and PrP^Sc^ typing.

## Materials and Methods

### Ethics Statements

Written informed consent for participation in research and all procedures for sample collection and experimental studies were in accordance with the 1964 Declaration of Helsinki and its later amendments and were approved by the Ethical Committee of “Fondazione IRCCS Istituto Neurologico Carlo Besta” (Milan, Italy). Tg(MHu2M)FVB-B5378 mice were housed in individually ventilated cages (2–5 mice per cage), daily fed, and provided with water. Lighting was on an automatic 12 h basis. Regular care was periodically performed for assessment of animal health. The animal facility is licensed and inspected by the Italian Ministry of Health. Current animal husbandry and housing practices comply with the Council of Europe Convention ETS123 (European Convention for the Protection of Vertebrate Animals used for Experimental and Other Scientific Purposes; Strasbourg, 18.03.1986), Italian Legislative Decree 116/92 (Gazzetta Ufficiale della Repubblica Italiana, 18 February 1992), and with the 86/609/EEC (Council Directive of 24 November 1986 on the approximation of laws, regulations, and administrative provisions of the Member States regarding the protection of animals used for experimental and other scientific purposes). The animal study, including its Ethics aspects, was approved by the Italian Ministry of Health (Permit Number: 258/2018-PR), and all efforts were made to minimize animals suffering.

### Mice and Human Genotyping

Murine genomic DNA was extracted from ear biopsies and analyzed by PCR using specific primers (5′-GAACTG AACCATTTCAACCGAG-3′ and 5′-AGAGCTACAGGTGGA TAACC-3′). Denaturation at 95°C for 5 min was followed by 35 cycles of amplification (95°C for 1 min, 58°C for 1 min, 72°C for 1 min). PCR products were resolved by agarose gel electrophoresis and analyzed for the presence of the transgene. Human DNA was extracted from peripheral blood mononuclear cells (PBMC). The coding sequence of *PRNP* gene was amplified by PCR using two pairs of primers: forward 5′-CAGAGAAGTACAGGGTGGCA-3′/reverse 5′-AATGTATGA TGGGCCTGCTCAT-3′ and forward 5′-CAACATGAAGCAC ATGGCTGGT-3′/reverse 5′-TAAAAGGGCTGCAGGTGGAT AC-3′. The amplified fragments were sequenced using the BigDye Terminator v1.1 Cycle Sequencing kit (Applied Biosystems) and analyzed on an ABI 3130xl Genetic Analyzer (Applied Biosystem).

### Collection of Biological Samples

A total of 65 OM samples were included in the study (see Table 1). In particular, 27 were collected from sCJD patients (MM = 13, MV = 8 and VV = 6), 2 from genetic CJD (gCJD) patients harboring the E200K mutation and 36 from patients with other neurodegenerative/neurological disorders (OND), including Alzheimer’s disease (AD = 3) (McKhann et al., 2011), Parkinson’s disease (PD = 7) (Postuma et al., 2015), frontotemporal dementia (FTD = 7) (Rascovsky et al., 2011), multiple system atrophy (MSA = 4) (Gilman et al., 2008), progressive supranuclear palsy (PSP = 7) (Höglinger et al., 2017), corticobasal degeneration (CBD = 6) (Alexander et al., 2014) and multiple sclerosis (MS = 2) (Table 1). OM samples were collected between the septum and the middle turbinate in the vault of the nose by brushing with a cotton swab, as previously described (De Luca et al., 2019). Cotton swabs were then immersed in saline solution and subjected to vigorous shaking to collect OM cells. Samples were then stored at −80°C until analyses. A total of 28 brains were included in the study. In particular, frozen samples of frontal cortex (*gyrus cinguli*) were collected from 26 sCJD patients (MM1 = 7, MV1 = 3, VV1 = 1, MM2-cortical = 3, MM2-thalamic = 2, MV2 = 6, VV2 = 4) and 2 non-CJD patients (AD = 1_129MM and FTD = 1_129MV). Sixteen of the sCJD brains (MM1 = 6, MV2 = 6, and VV2 = 4) belonged to patients who underwent OM collection (see Table 2 for details). The demografic data, genetic, instrumental and laboratory findings of all sCJD patients who underwent OM collection are reported in Table 3. The brains of the AD and FTD patients who donated OM samples were not collected.

**TABLE 1:** Summary of the OM samples included in the analysis.

Disease	129 *PRNP* genotype	Number of patients
Sporadic CJD	MM	13
	MV	8
	VV	6
Genetic CJD (E200K)	MM	1
	MV	1
Other neurodegenerative/neurological diseases: Alzheimer’s disease (AD), corticobasal degeneration (CBD), progressive supranuclear palsy (PSP), multiple system atrophy (MSA), Parkinson’s disease (PD), frontotemporal dementia (FTD), and multiple sclerosis (SM)	MM	14 (AD = 1, CBD = 2, FTD = 3, MSA = 2, PD = 3, PSP = 3)
	MV	16 (AD = 1, CBD = 2, FTD = 3, MSA = 1, PD = 3, PSP = 4, MS = 2)
	VV	6 (AD = 1, CBD = 2, FTD = 1, MSA = 1, PD = 1)

**TABLE 2:** Details of the olfactory mucosa (OM) and brain homogenates (BHs) analyzed.

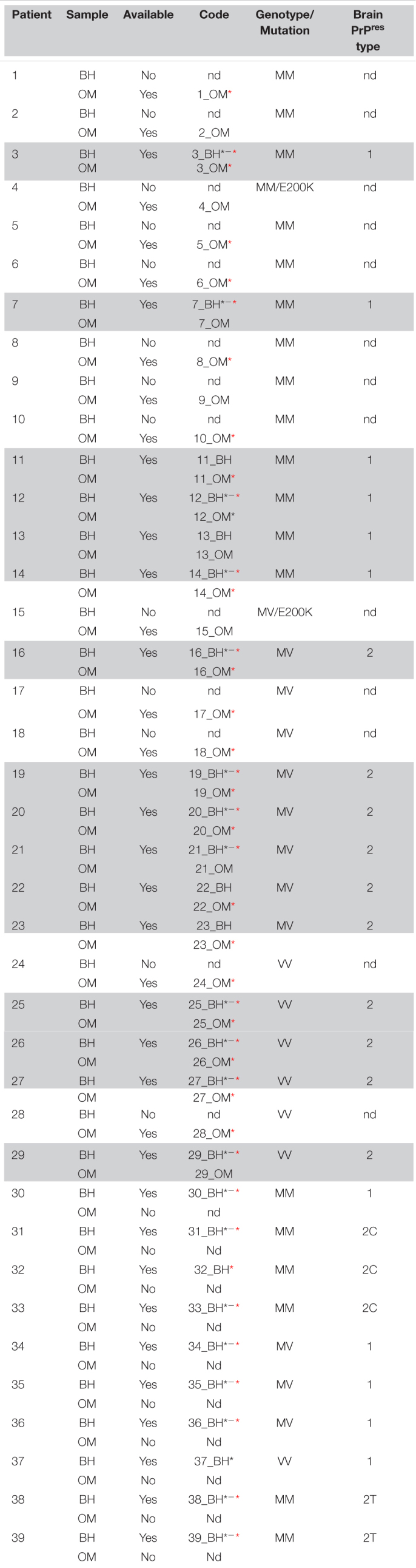

*Black asterisk indicates samples that were subjected raw to PK resistant analysis, while red asterisk refers to PMCA generated products that underwent similar evaluation; nd: not determined; 2T, MM2-thalamic; 2C, MM2-cortical. Gray color indicates patients whom brain and olfactory mucosa were collected from.*

**TABLE 3:** Summary of the demographic data, genetic, instrumental and laboratory findings of the sCJD patients who underwent OM collection.

Patient	Sex (Male)	*PRNP* 129	Type of PrP^res^	14.3.3	p-tau (pg/mL)	t-tau (pg/mL)	Mutation	Positive MRI (Y/N)	Dementia (Y/N)	EEG	Age at onset (years)	Disease duration at OM collection (months)	Time to death at OM collection (months)	Disease duration (months)	OM RT-QuIC results	CSF RT-QuIC results
1	+	MM	nd	Weakly positive	22	4,261		Y	Y	Refractory status epilepticus	57	2.9	17.4	20.3	+	+
2	-	MM	nd	nd	nd	nd		Y	Y	Triphasic	55	2.3	1.2	3.6	+	nd
3	-	MM	1	Positive	nd	30,150		Y	Y	Triphasic	42	5.0	29.2	34.2	+	nd
4	-	MM	nd	Positive	59	1,126	E200K	Y	Y	Slow waves	54	9.1	1.0	10.1	+	nd
5	+	MM	nd	Negative	27	292		Y	Y	No periodic	45	19.2	23.8	43.0	+	nd
6	+	MM	nd	Positive	118	1,698		Y	Y	Normal	65	7.8	27.9	35.7	+	+
7	+	MM	1	Positive	41	6,220		Y	Y	Triphasic	55	2.0	1.4	3.4	+	nd
8	+	MM	nd	Weakly positive	nd	1,187		Y	Y	Triphasic	42	1.0	48.4	49.4	+	+
9	-	MM	nd	Weakly positive	39	879		Y	Y	Triphasic	78	7.0	5.8	12.8	+	+
10	+	MM	nd	Positive	42	7,934		Y	Y	Triphasic	70	1.8	0.3	2.0	+	+
11	-	MM	1	Positive	86	2,633		Y	Y	Triphasic	59	7.3	22.1	29.4	+	+
12	-	MM	1	Positive	46	>2,400		Y	Y	Triphasic	74	0.9	1.2	2.0	+	+
13	+	MM	1	Positive	25	>2,400		Y	Y	Triphasic	72	2.3	2.3	4.6	+	+
14	+	MM	1	Positive	31	>2,400		Y	Y	Triphasic	65	1.2	0.7	1.9	+	+
15	-	MV	nd	nd	nd	nd	E200K	N	Y	Slow waves	71	37.3	6.7	44.0	+	nd
16	+	MV	1	Negative	38	680		Y	Y	Triphasic	77	14.0	10.6	24.5	+	+
17	-	MV	nd	Weakly positive	53	3,057		Y	Y	Slow waves	65	12.9	10.3	23.2	+	nd
18	+	MV	nd	Weakly positive	nd	nd		nd	nd	nd	nd	nd	5.9	nd	+	nd
19	+	MV	1	Positive	65	1,788		Y	Y	No periodic	69	8.0	3.2	11.2	+	+
20	-	MV	2	nd	80	2,101		Y	Y	Slow waves	62	4.4	24.5	28.9	+	+
21	+	MV	2	Weakly positive	16.7	1,076		Y	Y	Slow waves	64	5.1	4.8	9.9	+	+
22	-	MV	2	Weakly positive	76	1,986		Y	Y	Slow waves	74	7.4	7.5	14.8	+	+
23	+	MV	2	Negative	55	1,905		Y	Y	Triphasic	79	8.2	4.2	12.4	+	+
24	-	VV	nd	Positive	92.8	18,470		Y	Y	No periodic	61	4.6	6.5	11.1	+	nd
25	+	VV	2	nd	nd	nd		Y	Y	Normal	66	5.7	3.5	9.3	+	nd
26	-	VV	2	Positive	75	15,575		Y	Y	No periodic	55	8.5	0.6	9.1	+	nd
27	-	VV	2	Positive	81	15,750		Y	Y	Slow waves	57	3.3	1.2	4.5	+	nd
28	-	VV	nd	Positive	nd	5,026		Y	Y	Triphasic	66	1.5	1.1	2.6	+	nd
29	+	VV	2	Positive	61	>2,400		Y	N	Slow waves	40	2.0	3.0	5.0	+	+

### Preparation of Human Brain Homogenates for Biochemical, RT-QuIC and PMCA Analyses

Frozen brain samples (sCJD, AD and FTD) were homogenized at 10% (weight/volume, w/v) in lysis buffer (100 mM sodium chloride, 10 mM ethylenediaminetetraacetic acid tetrasodium salt (EDTA), 0.5% Non-idet P-40, 0.5% sodium deoxycholate, 10 mM Tris, pH 7.4). Brain homogenates (BHs) were then centrifuged at 4°C, 800 × *g*, for 1 min to remove cellular debris. The supernatant was then subjected to different analyses. In particular, 10 μL was treated with PK and subjected to Western blot (Wb) analysis to verify the presence of PrP^res^ and determine its biochemical properties (glycoform ratio and typing). Fifty μL was used to perform PK resistance analysis (as detailed in the section “Proteinase-K Resistant Profile of Brain Homogenates and PMCA Generated Products”). Twenty μL of selected BHs was used to perform Peptide-*N*-Glycosidase F (PNGase F) experiments (as detailed in the section “PNGase F Treatment of Human Brain Homogenates”). Each BH was diluted at 10^–4^ in phosphate buffer saline (PBS) (volume/volume, v/v) and 2 μL of this dilution was subjected to RT-QuIC analysis (as detailed in the section “RT-QuIC Analysis of Brain Homogenates and Olfactory Mucosa”). Finally, each BH was diluted from 10^–3^ to 10^–12^ in PBS (v/v) and every dilution was subjected to PMCA analysis (as detailed in the section “PMCA Analysis of Brain Homogenates and Olfactory Mucosa”).

### Preparation of Olfactory Mucosa for RT-QuIC and PMCA Analyses

Olfactory mucosa samples were thawed and centrifuged at 4°C, 800 × *g*, for 20 min. The saline solution was removed and approximately 2 μg of the pellet was collected using an inoculating loop, and dissolved in 25 μL of PBS (pH 7.4, Sigma). Ten μL of this solution was used to perform PMCA analysis (as detailed in the section “PMCA Analysis of Brain Homogenates and Olfactory Mucosa”). Two μL was further diluted into 18 μL of PBS and 2 μL of this latter dilution was used to perform RT-QuIC analysis (as detailed in the section “RT-QuIC Analysis of Brain Homogenates and Olfactory Mucosa”).

### Production of Recombinant PrP Proteins for RT-QuIC Analysis and Quantitative PMCA

Full-length human PrP with methionine at position 129 (recHuPrP_23–231_) and truncated Syrian hamster PrP (recHaPrP_90–231_) constructs were expressed in Escherichia coli BL21 (DE3) cells (Stratagene). One hundred mL of overnight culture was inoculated into Luria-Bertani (LB) medium complemented with 100 μg/mL ampicillin. At 0.6 OD600 the expression of the constructs was induced with 0.8 mM of isopropyl b-D galactopyranoside (IPTG). Cells were grown at 30°C for 12 h and then lysed in 25 mM Tris–HCl, 5 mM EDTA, 0.8% Triton X-100, pH 8.0 with fresh added 1 mM phenylmethylsulfonyl fluoride (PMSF) using PandaPLUS 2000. Inclusion bodies containing the recombinant proteins were washed several times in bi-distilled water and then dissolved in 8 M guanidine hydrochloride (GdnHCl) before loading onto a pre-equilibrated HiLoad 26/60 Superdex 200 pg column (Cytiva) and eluted in 5 M GdnHCl, 25 mM Tris–HCl, 5 mM EDTA, pH 8.0 at a flow rate of 2 mL/min. Protein refolding was performed by dialysis against 20 mM sodium acetate, pH 5.5 using Spectrapor membrane. Purified proteins were analyzed by SDS-polyacrylamide gel electrophoresis under reducing conditions, dialyzed against phosphate buffer, pH 5.8 and stored at −80°C. All salts used were from Sigma-Aldrich. RecHaPrP_90–231_ was used as a reaction substrate for RT-QuIC analyses, while recHuPrP_23–231_ was used for quantitative PMCA.

### RT-QuIC Analysis of Brain Homogenates and Olfactory Mucosa

RecHaPrP_90–231_ was thawed and filtered through a 100 kDa Nanosep centrifugal device (Pall Corporation). RT-QuIC reaction mix was composed of 10 mM PBS (pH 7.4), 150 mM NaCl, 0.13 mg/ml recHaPrP_90–231_, 1 mM EDTA, 0.002% SDS and 10 μM thioflavin T (ThT). Ninety−eight μL of the reaction mix was placed in a black 96-well optical flat bottom plate (Thermo Scientific) and supplemented with 2 μl of OM or BH (prepared as previously described). Each sample has been analyzed in quadruplicate. The plate was sealed with a sealing film (Thermo Scientific) and underwent intermittent cycles of shaking (1 min at 600 rpm, double orbital) and incubation (1 min) at 55°C using the fluorescence microplate reader OPTIMA (BMG Labtech). ThT fluorescence was measured every 30 min (wave-lengths: excitation 450 ± 10 nm; emission 480 ± 10 nm). A sample was considered positive if the fluorescence value of at least 2 out of 4 replicates was higher than 10,000 arbitrary units (AU) before the threshold of time set at 60 h, as described (Franceschini et al., 2017).

### Preparation of the Substrate for PMCA Analysis

The brains of Tg(MHu2M)FVB-B5378 mice (Telling et al., 1994), from now on referred to as TgHuMM mice, were homogenized at 10% (w/v) in conversion buffer (PBS 1X containing 150 mM sodium chloride and 1% Triton X-100) supplemented with cOmplete Mini EDTA-free protease inhibitor cocktail (Roche) and used as a PMCA reaction substrate. To increase PMCA efficiency, 0.135 M sodium tripolyphosphate, 6 mM EDTA, 100 μg/mL heparin, 0.05% digitonin (Sigma), and 3 Teflon beads were used to supplement the substrate.

### PMCA Analysis of Brain Homogenates and Olfactory Mucosa

Ten μL of each dilution of BH or OM sample (prepared as previously described) was added to 90 μL of PMCA substrate, transferred into 0.2 mL PCR tubes and subjected to amplification using a Qsonica Q700 sonicator. PMCA consisted of intermittent cycles of incubation (29 min and 20 s) and sonication (40 s set at 260–280 W). After 48 h of reaction (considered as a round of amplification), 10 μL of the amplified material was added to 90 μL of freshly prepared PMCA substrate and an additional round of amplification was performed. In total, each sample (OM or BH) was subjected to 6 (or in specific cases to 7) PMCA rounds. To avoid any contamination, PMCA substrates were prepared under rigorous prion-free conditions and the sonicator horn was periodically decontaminated with 4 M Gdn-HCl (overnight). To monitor the contamination and the potential *de novo* generation of prions, appropriate negative controls were included in each PMCA round. All the analyses have been repeated three times by three different operators to check the reproducibility of the results. Amplified products obtained from BH were named BH_PMCA while those obtained from OM were named OM_PMCA.

### Proteinase-K Digestion of Brain Homogenates and PMCA Generated Products

Ten μL of (i) BHs (sCJD, AD and FTD), (ii) BH_PMCA and (iii) OM_PMCA were treated with 50 μg/mL of proteinase K (PK, Invitrogen) for 1 h at 37°C under shaking (550 rpm) before Wb analysis and immunoblotting with 6D11 antibody (1:5,000, epitopes 93–109, Covance). Ten μL of BHs were digested with 400 μg/mL of PK for Wb analysis and immunoblotting with 12B2 antibody (1:8,000, epitopes 89–93).

### Proteinase-K Resistant Profile of Brain Homogenates and PMCA Generated Products

The PK resistant profile of the samples marked with red and/or black asterisks in Table 2 has been assessed. In particular, the black asterisk refers to the analysis of raw BHs (22 samples: MM1 = 5, MM2C = 3, MM2T = 2, MV1 = 3, MV2 = 4, VV1 = 1, VV2 = 4) while the red asterisk refers to the analysis of BH_PMCA (20 samples: MM1 = 5, MM2C = 2, MM2T = 2, MV1 = 3, MV2 = 4, VV2 = 4) and OM_PMCA [21 samples: MM = 9 (4 MM1, 5 unknown PrP^res^ typing), MV = 7 (5 MV2, 2 unknown PrP^res^ typing), VV = 5 (3 VV2, 2 unknown PrP^res^ typing)]. Each sample was treated with five increasing concentrations of PK (50, 250, 500, 1,000, and 1,500 μg/mL) and incubated for 1 h at 37°C under shaking (500 rpm). The enzymatic activity was stopped by boiling the sample for 10 min in loading buffer (Bolt™ LDS Sample Buffer and Dithiothreitol (DTT), Thermo Scientific) before Wb analyses. Densitometric quantification of PrP^res^ bands was performed as described in the section “Statistical Analysis.”

### Sodium Dodecyl Sulfate-Polyacrylamide Gel Electrophoresis and Western Blotting

Proteinase K digested samples were loaded into 12% BisTris plus gels (Thermo Scientific) and subjected to electrophoresis analysis under denaturing conditions (SDS-PAGE). Samples that did not require PK treatment (e.g. recHuPrP_23–231_) were immediately supplemented with loading buffer (Bolt™ LDS Sample Buffer 4X and DTT 10X, ThermoScientific) and boiled for 10 min before SDS-PAGE. Proteins were then transferred into polyvinylidene difluoride membranes (PVDF, Millipore) and incubated with non-fat dry milk (Santa Cruz) for 1 h at room temperature. PVDF membrane was probed using the monoclonal anti-PrP antibody 6D11 and then incubated with Fab fragment anti-mouse IgG conjugated with horseradish peroxidase (HRP) (GE). Blots were developed with a chemiluminescent system (ECL Prime, GE Healthcare Amersham). When required, densitometric quantification of PrP^res^ bands was performed as described in the section “Statistical Analysis.”

### PNGase F Treatment of Human Brain Homogenates

Twenty μL of selected BHs (3_BH, 16_BH, 19_BH, 25_BH, 26_BH, see below for details) was digested with 100 μg/mL of PK for 1 h at 37°C. PNGase F treatment was performed according to the manufacturer’s instructions (New England Biolabs PNGase F P0704S). Briefly, after digestion samples were supplemented with 4 μL di 10X glycoprotein denaturing buffer and boiled for 10 min. Then, samples were supplemented with 4 μL of reaction buffer (10X), 4 μL of NP-40 (10%), 4 μL of PNGase F and 4 μL of PBS and incubated overnight at 37°C. After incubation, 20 μL of LDS-PAGE loading buffer (Sample buffer 4X and DTT 10X, Thermo Scientific) was added to the samples that were boiled for 10 min. Finally, four serial dilutions (1:2) of each sample were prepared, loaded into 12% BisTris plus gels (Thermo Scientific) and subjected to Wb analysis.

### Quantitative PMCA Analysis

Quantitative PMCA (qPMCA) was performed as previously described (Chen et al., 2010; Moda et al., 2014). In particular, we have selected 5 patients (3, 16, 19, 25 and 26) with known sCJD subtypes (MM1 = 1, MV2 = 2, VV2 = 2) in which prions were efficiently amplified from both the OM and the corresponding brain samples (see Table 2 for details). To this aim, known amounts of recHuPrP_23–231_ (8, 6, 4, 2, and 1 ng) were analyzed by SDS-PAGE together with the dilutions of the PNGase treated BHs, to estimate their specific PrP^res^ content. Samples were immunoblotted with the 6D11 antibody and the intensities of each recHuPrP_23–231_ band (representing known amounts of protein) were compared with those of the PrP^res^ present in each BH dilution. In this way, we have estimated the concentration of PrP^res^ in every sCJD-BH. Densitometric analysis of all resulting PrP bands was performed as described in the section “Statistical Analysis.”

### Statistical Analysis

Densitometric analysis of Western blot bands were carried out using ImageJ software (1.48v). Graphic representations of densitometric analysis were performed using the Prism software (GraphPad v7.0.5). PK resistance profiles of PrP^res^ derived from BH, BH_PMCA and OM_PMCA were analyzed through repeated analysis of variance (ANOVA). Associations between variables were investigated through *t*-test or Mann–Whitney test and Fisher exact test, as appropriate.

## Results

### RT-QuIC Analysis of Olfactory Mucosa and Brain Homogenates of Sporadic Creutzfeldt-Jakob Disease Patients and Controls

RT-QuIC analysis of the OM showed that all samples collected from sporadic and genetic CJD patients (*n* = 29), regardless of the polymorphism at *PRNP*129, induced an efficient seeding activity for recHaPrP_90–231_ before 35 h. The reaction was stopped at 60 h and none of the OM collected from patients with other neurodegenerative/neurological disorders (OND) induced any seeding activity (Supplementary Figure 1A). We have then subjected to RT-QuIC analysis the brains of the 16 sCJD patients who underwent OM collection during life. Also in this case, regardless of the polymorphism at *PRNP*129, all sCJD brains induced an efficient seeding activity while AD and FTD brains did not (Supplementary Figure 1B).

### Western Blot Analysis of Brain Homogenates

The brain homogenates (BHs) of 26 sCJD patients were analyzed by Western blot (Wb) to confirm prion disease diagnosis and determine the biochemical properties of PrP^res^. According to the polymorphism at *PRNP*129 and the PrP^res^ properties, samples were classified in the following groups: (i) MM1 = 7, (ii) MV1 = 3, (iii) VV1 = 1, (iv) MM2-cortical = 3 or (v) MM2-thalamic = 2 (differentiated by histopathological analysis), (vi) MV2 = 6 and (vii) VV2 = 4 (Supplementary Figures 2A–E). Sixteen of these brains belonged to patients whom the OM has been collected from: MM1 = 6, MV2 = 6, and VV2 = 4 (see Table 2 for details). The BHs of a patient with AD (MM) and a patient with FTD (MV) were included as negative controls and no PrP^res^ was detected (Supplementary Figure 2E). The same samples have been immunoblotted with the 12B2 antibody, which recognizes type 1 PrP^res^, and in 6 MV2 and 4 VV2 samples we could detect the co-occurrence of type 1 and type 2 PrP^res^ (Supplementary Figures 2F–J).

### PMCA Analysis of Sporadic Creutzfeldt-Jakob Disease Brain Homogenates

**FIGURE 1:**
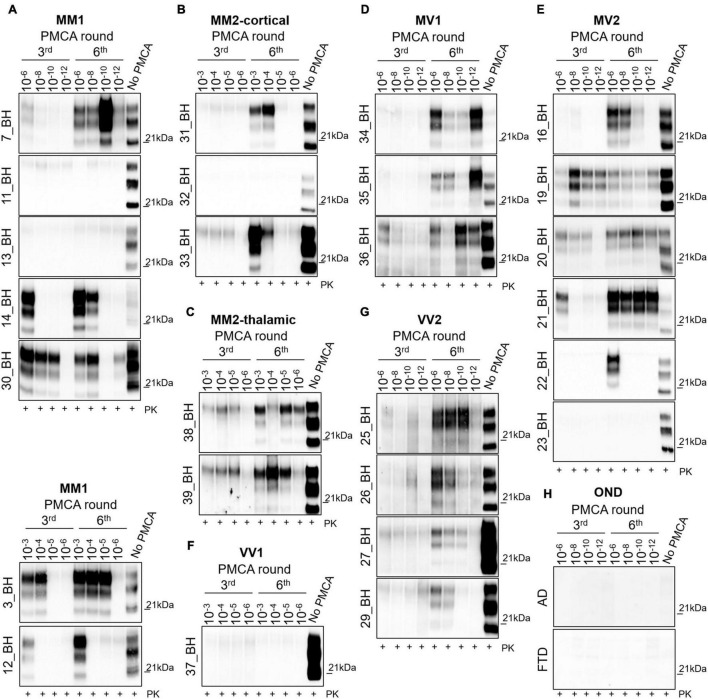
Analysis of brain homogenates by PMCA. Wb of the 3*^rd^* and 6*^th^* PMCA rounds are shown. After 6 rounds of amplification, PrP^res^ was detected in the brains of **(A)** 5/7 MM1, **(B)** 2/3 MM2C, **(C)** 2/2 MM2T, **(D)** 3/3MV1, **(E)** 5/6 MV2, **(F)** 0/1 VV1, and **(G)** 4/4 VV2, although with variable efficiency. **(H)** No PrP^res^ was amplified from the brain of OND patients (AD and FTD). **(A–C)** In particular, type 2 PrP^res^ with a prevalence of the di-glycosylated species was generated by the BHs of 4/7 MM1, 2/3 MM2C and 2/2 MM2T patients, except for one MM1 patient (7_BH) in which the presence of a type 1 PrP^res^ with an equal representation of the di- and mono-glycosylated band was observed. **(D–G)** In contrast, type 1 PrP^res^ was generated by BHs of 3/3 MV1, 5/6 MV2 patients and 4/4 VV2 patients. Except for 2 VV2 samples (25_BH and 26_BH), all amplified PrP^res^ were characterized by a prevalent di-glycosylated band. Numbers in the right of each Wb indicate the molecular weight marker.

To test the amplification efficiency of sCJD prions, we have subjected all 26 sCJD BHs to PMCA analysis (see Table 2). Regarding the MM1 subtype, prions were detected in 5/7 BHs at the 6^th^ round. In particular, in three BHs (7, 14 and 30) PrP^res^ was amplified with high efficiency (dilutions 10^–8^–10^–12^) while in two BHs (3 and 12) the amplification was less efficient and PrP^res^ was detected only in lower dilutions (10^–3^–10^–5^). No PrP^res^ was detected in two BHs (11 and 13) even after 6 rounds of amplification (Figure 1A). Notably, except for 7_BH, all amplified PrP^res^ switched from type 1 to type 2 and densitometric analysis revealed the presence of higher levels of the di-glycosylated species (Supplementary Figure 3A). Contrarily, in 7_BH the amplified PrP^res^ did not switch typing and the di-glycosylated and mono-glycosylated PrP bands were equally expressed (Supplementary Figure 3A). In the case of cortical and thalamic MM2 subtypes (MM2C and MM2T, respectively), PrP^res^ was amplified with low efficiency (dilutions 10^–3^–10^–6^) in 2/3 MM2C BHs (31 and 33) (Figure 1B) and all MM2T (38 and 39) (Figure 1C). No PrP^res^ was detected in 32_BH (MM2C) even after 6 rounds of amplification. Prions amplified from these BHs maintained type 2 profile but their glycoform ratios differed from those of the original strains and a predominant di-glycosylated band was observed (Supplementary Figure 3A). Regarding the MV subtypes, we could efficiently amplify PrP^res^ (dilutions 10^–6^–10^–12^) in all MV1 BHs (34, 35 and 36) (Figure 1D) and 5/6 MV2 (16, 19, 20, 21 and 22) (Figure 1E). No PrP^res^ was detected in 23_BH (MV2) even after 6 rounds of amplification. In both subtypes, the amplification led to the formation of type 1 PrP^res^ with the di-glycoyslated species predominant over the others (Supplementary Figure 3B). Thus, prions amplified in MV1 samples maintained the typing of the original inocula while those amplified in MV2 switched from 2 to 1. Finally, we efficiently amplified prions (dilutions 10^–6^–10^–12^) in all VV2 BHs (25, 26, 27 and 29) that showed type 1 PrP^res^ but in two of them (27 and 29) the di-glycoyslated species prevailed while in the other two (25 and 26) the di- and mono-glycosylated bands were equally represented (Supplementary Figure 3C). Hence, also in this case, amplified PrP^res^ did not maintain the typing of the original strain (Figure 1G). No PrP^res^ was amplified in the only available VV1 sample (37_BH) even after 6 rounds of amplification (Figure 1F). As expected, no PrP^res^ was amplified from the brains of AD or FTD patients (Figure 1H). Regardless of the co-occurrence of type 1 and type 2 PrP^res^ in all MV2 and VV2 BHs, the amplified products acquired similar biochemical properties thus suggesting that the PMCA was able to generate a distinctive prion isolate (Polymenidou et al., 2005; Notari et al., 2007; Cali et al., 2020). Interestingly, these particular switches of PrP^res^ typing after PMCA analysis of sCJD brain homogenates were recently described by the group of Bougard (Bélondrade et al., 2021).

### PMCA Analysis of Sporadic Creutzfeldt-Jakob Disease Olfactory Mucosa

**FIGURE 2:**
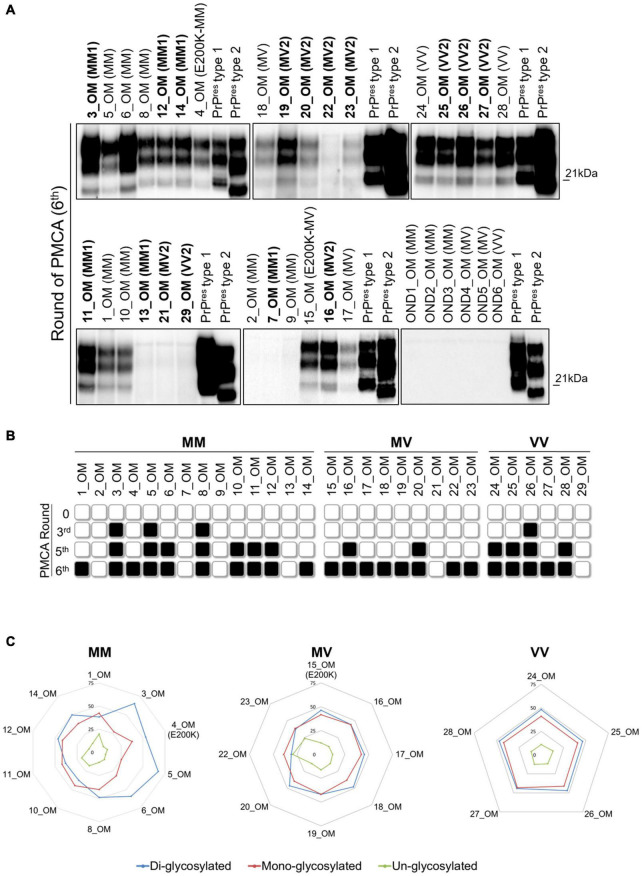
Analysis of olfactory mucosa samples by PMCA. **(A)** PrP^res^ detection in OM of 10/14 MM samples (including 4 MM1, 1 E200K and 5 MMunk), 8/9 MV samples (including 5 MV2, 1 E200K and 2 MVunk) and 5/6 VV samples (including 3 VV2 and 2 VVunk). Samples with known PrP^res^ typing are written in bold. No PrP^res^ was found in the OM of patients with OND (AD: OND1, OND4 and OND6; Parkinson’s disease: OND2; Corticobasal degeneration: OND3 and OND5). Numbers in the right of each Wb indicate the molecular weight marker. **(B)** Schematic representation of the PMCA rounds at which PrP^res^ was detected in each OM sample. Black and white boxes indicate the presence or absence of PrP^res^, respectively. **(C)** Radar plots showing the PrP^res^ predominant species of OM_PMCA samples. Numerical scale in each radar plot indicates the mean density of the PrP^res^ isoform.

To test whether our PMCA protocol could also amplify prions in the OM of sCJD patients, we have analyzed 65 samples collected from 27 sCJD, 2 gCJD, and 36 OND patients (see Table 1 for details). From the whole group of MM samples (*n* = 14), we could amplify PrP^res^ in 10 OM (71.4%), including 4 MM1, 1 E200K and 5 samples with unknown PrP^res^ typing (MMunk). Two MM1 and 2 MMunk remained negative (Figures 2A,B). The majority of the PrP^res^ amplified in this group of OM were of type 1 and characterized by an equal representation of the di- and mono-glycosylated species (1, 4, 8, 10, 11, 12 and 14). In contrast, three of them (3, 5 and 6) were of type 2 with a prevalence of the di-glycosylated species (Figures 2A–C and Supplementary Figure 3A). Considering only the MM1 samples, we were able to amplify prions in 4/6 OM (67%). Interestingly, 3 OM that generated type 1 PrP^res^ (11, 12 and 14) had the corresponding BHs that amplified type 2 PrP^res^, except for 11_BH that remained negative (Figure 1). The remaining OM (number 3) and the corresponding BH (number 3 in Figure 1) amplified type 2 PrP^res^. Therefore, the biochemical properties of MM prions (in terms of typing or glycoform ratio) were not faithfully maintained after the amplification. Surprisingly, even the PrP^res^ amplified from BH and OM of the same sCJD patient often possessed distinct biochemical features (e.g., in patient 12 type 1 PrP^res^ was amplified from BH while type 2 PrP^res^ was amplified from the corresponding OM) (Figures 1A, 2A and Supplementary Figure 3A). From the whole group of MV samples (*n* = 9), we were able to amplify PrP^res^ in 8 OM (88.8%), including 5 MV2, 1 E200K and 2 samples with unknown PrP^res^ typing (MVunk), while 1 MV2 remained negative (Figures 2A,B). All amplified products in this group of OM (15, 16, 17, 18, 19, 20, 22 and 23) were characterized by type 1 PrP^res^ with an equal representation of the di- and mono-glycosylated species (Figures 2A–C and Supplementary Figure 3B). Considering only the MV2 samples, we were able to amplify prions in 5/6 OM (83.3%). Interestingly, their corresponding BHs amplified type 1 PrP^res^ but with a distinct glycoform ratio that was characterized by predominant di-glycosylated bands, instead (Supplementary Figure 3B). Finally, from the whole group of VV samples (*n* = 6), we were able to amplify PrP^res^ in 5 OM (83.3%), including 3 VV2 and 2 samples with unknown PrP^res^ typing (VVunk), while 1 VV2 remained negative (Figures 2A,B). All amplified products in this last group of OM (24, 25, 26, 27 and 28) showed type 1 PrP^res^ with an equal representation of the di- and mono-glycosylated species (Figures 2A–C and Supplementary Figure 3C). Considering only the VV2 samples, we were able to amplify prions in 3/4 OM (75%). Their corresponding BHs also amplified type 1 PrP^res^ but their glycoform ratio was characterized by a higher representation of the di-glycosylated band (27_BH and 29_BH) or an equal representation of di- and mono-glycosylated species (25_BH and 26_BH) (Supplementary Figure 3C). We have then evaluated at which PMCA round the OM samples showed detectable PrP^res^ and found that: 3 MM (3, 5 and 8) and 1 VV (number 26) showed PrP^res^ at the 3^rd^ PMCA round; 4 MM (6, 10, 11 and 12), 2 MV (16 and 20), and 3 VV (24, 25 and 28) showed PrP^res^ at the 5^th^ PMCA round; while 3 MM (1, 4 and 14), 6 MV (15, 17, 18, 19, 22 and 23), and 1 VV (number 27) showed PrP^res^ at the 6^th^ PMCA round (Figure 2B). No PrP^res^ was detected in the 36 OM samples of OND patients collected at the 6^th^ round (Figure 2A and Supplementary Figure 4). The biochemical properties of PrP^res^ detected in BH, BH_PMCA and OM_PMCA are summarized in Supplementary Table 1. All samples were analyzed at least three time by three different operators and analogous results were always obtained, thus confirming the reproducibility of the analytical procedures and the reliability of our findings.

### Evaluation of the Biochemical Properties of Protein Misfolding Cyclic Amplification Generated Products

Considering that the PrP^res^ amplified from BH and OM did not retain the specific features of the original prions and that, in most of the cases, they also differed between samples belonging to the same patients, we decided to test whether and to what extent the PMCA could have altered the prion properties during the amplification.

**FIGURE 3:**
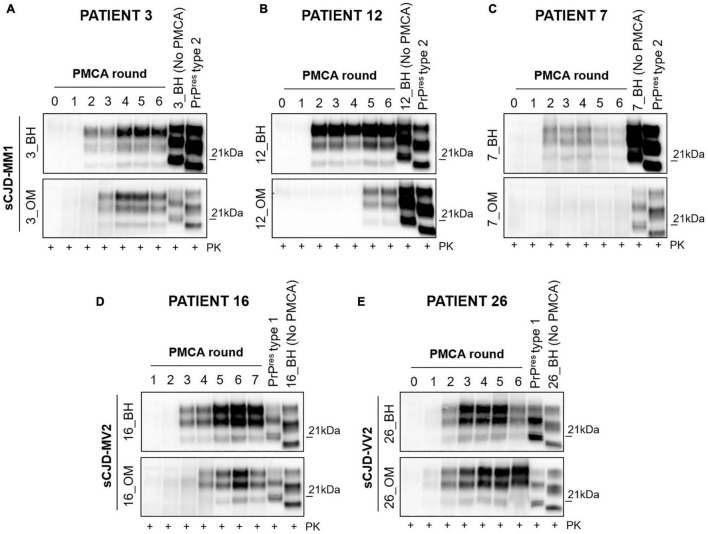
Analysis of the biochemical properties of PrP^res^ generated at each round of amplification from BH and OM of MM1, MV2 and VV2 patients. From the first appearance to the end of the amplification the PrP^res^ maintained both glycoform ratio and typing. **(A)** The BH and OM of patient 3 generated type 2 PrP^res^ with a predominant di-glycosylated species; **(B)** BH and OM of patients 12 generated type 2 and type 1 PrP^res^, respectively, that were also characterized by distinct glycoform ratios; **(C)** OM of patient 7 remained negative while BH gave rise to type 1 PrP^res^ with an equal representation of the di- and mono-glycosylated species; **(D)** BH and OM of patient 16 generated type 1 PrP^res^ with different glycoform ratios. **(E)** BH and OM of patient 26 generated type 1 PrP^res^ with similar levels of the di- and mono-glycosylated isoforms.

Initially, we have evaluated if the original prions might have undergone processes of selection and adaptation during the amplification, finally leading to the onset of different isolates. To this aim, we have selected 3 MM1 patients (number 3, 7 and 12), 1 MV2 patient (number 16), and 1 VV2 patient (number 26) whose PMCA results obtained from BH and OM were the most representative for their specific sCJD subgroup (Figure 3). In particular, we have observed that (i) BH and OM of patient 3 generated type 2 PrP^res^ with a prevalent di-glycosylated species (Figure 3A and Supplementary Figure 3A); (ii) BH and OM of patient 12 gave rise to type 2 and type 1 PrP^res^, respectively, with glycoform ratios that differed from each other (Figure 3B and Supplementary Figure 3A); (iii) BH of patient 7 generated type 1 PrP^res^ with an equal representation of the di- and mono-glycosylated species while the OM remained negative (Figure 3C and Supplementary Figure 3A); (iv) BH and OM of patient 16 generated type 1 PrP^res^ with a prevalent di-glycosylated band (Figure 3D and Supplementary Figure 3B) and (v) BH and OM of patient 26 generated type 1 PrP^res^ with the di- and mono-glycosylated isoforms similarly represented (Figure 3E and Supplementary Figure 3C). Unfortunately, we could not perform similar evaluations in MM2C, MM2T, MV1 and VV1 patients because their OM were not available. Figure 3 shows that from the round at which the amplified PrP^res^ could be detected by Wb, significant changes in their biochemical profiles were not observed. However, we do not know whether an adaptation process might have occurred in the first rounds of PMCA where the amplified prions could not yet be visualized.

**FIGURE 4:**
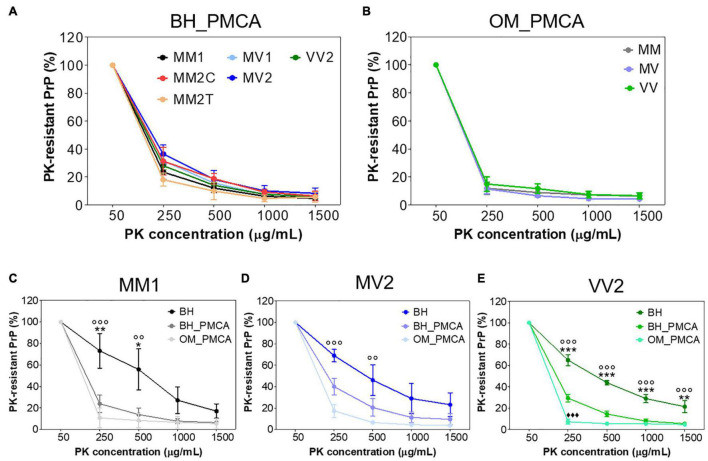
PK resistance analysis of BH and their amplified products collected at the 6^th^ PMCA round. **(A)** PK resistant profiles of all sCJD brain amplified products (BH_PMCA) did not show statistically significant differences. **(B)** PK resistant profiles of all sCJD OM amplified products (OM_PMCA) did not show statistically significant differences. **(C)** PK resistant profiles of BH, BH_PMCA and OM_PMCA of MM1 patients showing statistically significant differences only between BH and BH_PMCA or BH and OM_PMCA. **(D)** PK resistant profiles of BH, BH_PMCA and OM_PMCA of MV2 patients showing statistically significant differences only between BH and OM_PMCA. **(E)** PK resistant profiles of BH, BH_PMCA and OM_PMCA of VV2 patients showing statistically significant differences between BH, BH_PMCA and OM_PMCA. Statistical analyses: repeated measure analysis of variance (ANOVA); BH vs. BH_PMCA: *p < 0.05, **p < 0.01, ***p < 0.001, BH vs. OM_PMCA: °°*p* < 0.01, °°°*p* < 0.001 and BH_PMCA vs. OM_PMCA ^◆◆◆^*p* < 0.001; error bars: ± standard error of the mean [SEM].

We have then evaluated whether the sensitivity to PK digestion of each sCJD prion was lost after the amplification or retained instead. First of all, we have treated all BHs and their products of amplification (BH_PMCA) collected at the 6^th^ PMCA round with increasing concentrations of PK and found that, in the case of MM2C, MM2T and VV2 the amplified PrP^res^ were significantly more sensitive to digestion than the corresponding original strains [repeated measure analysis of variance (ANOVA): *p* = 0.0001, *p* = 0.0103, *p* < 0.0001, respectively]. In the case of MM1, MV1 and MV2, these differences were still present but they were not statistically significant (*p* = 0.0805, *p* = 0.0366 and *p* = 0.1200, respectively) (Supplementary Figure 5). Notably, all PrP^res^ amplified from BHs showed similar PK resistance profile (*p* = 0.4137) (Figure 4A).

We have then treated the OM amplified samples (OM_PMCA) with the same concentrations of PK and found that, if combined together, the amplified PrP^res^ were significantly less resistant to PK with respect to those of BH_PMCA (*p* < 0.0001). Also in this case, the PK resistance profiles of OM_PMCA prions were comparable between each other (*p* = 0.9616) (Figure 4B). We have then performed additional analyses by considering only samples (BH, BH_PMCA and OM_PMCA) collected from 9 autopsied cases: 3 MM1 (number 3, 12 and 14), 3 MV2 (number 16, 19 and 20) and 3 VV2 (number 25, 26 and 27). The results of this analysis are reported in Figure 4 and show that the PrP^res^ present in BH, BH_PMCA and OM_PMCA of VV2 possessed significantly different PK resistance profiles between each other (*p* < 0.0001) (Figure 4E). In the case of MM1 patients, statistically significant differences in the PK resistance profiles were observed between BH and BH_PMCA (*p* < 0.0001) or BH and OM_PMCA (*p* < 0.0001) but not between BH_PMCA and OM_PMCA (*p* = 0.6004). Regarding MV2 patients, we have observed statistically significant differences between the PK resistance profiles of BH and OM_PMCA (*p* = 0.0018) but not between BH and BH_PMCA (*p* = 0.1183) or BH_PMCA and OM_PMCA (*p* = 0.2492) (Figures 4C,D). Taken together these data indicate that although PMCA could amplify, with variable efficiency, PrP^Sc^ from the majority of BH and OM samples, the features of the original sCJD prions were altered thus hampering the possibility of their recognition.

### Estimating Prion Concentration in the Olfactory Mucosa of Sporadic Creutzfeldt-Jakob Disease MM1, MV2 and VV2 Patients by Means of Quantitative PMCA

**FIGURE 5:**
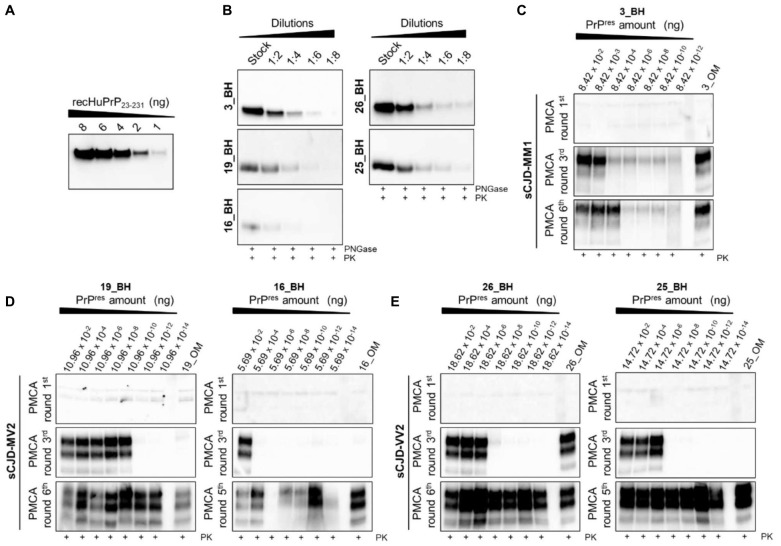
Quantitative PMCA (qPMCA) for estimating PrP^res^ concentration in OM samples of sCJD patients. **(A)** Serial dilutions of recombinant full-length human PrP (recHuPrP_23–231_) were used to estimate prion concentration in the brain of sCJD patients. **(B)** Serial dilutions of sCJD brain homogenates subjected to PK and PNGase treatments before Wb analysis. Quantitative PMCA to estimate PrP^res^ concentration in OM of **(C)** MM1, **(D)** MV2, and **(E)** VV2 patients. Specific rounds at which every OM PrP^res^ was detected (3^rd^ for the MM1 and one VV2, 5^th^ for one MV2 and one VV2, and 6^th^ for one MV2) are shown.

Through quantitative PMCA, we were able to estimate for the first time the concentration of prions in the OM samples of sCJD patients. Particularly, we have included one MM1 patient (patient 3), two MV2 patients (patient 16 and patient 19) and two VV2 patients (patient 25 and patient 26). Quantitative PMCA was performed following the protocol previously published by some of the authors of this manuscript (Chen et al., 2010; Moda et al., 2014; Redaelli et al., 2017). A highly accurate estimation of OM prions was made possible thanks to the availability of the corresponding brain samples that were used to calibrate the PMCA reactions. At the beginning, we have determined the PrP^res^ concentration in the brain of each sCJD patient. Brains were digested with PK treated with PNGase and analyzed by Wb (Figure 5B) along with known concentrations of human recombinant PrP (Figure 5A). In this way, we calculated that the PrP^res^ concentrations in 10 μL of the brains were as follow: patient 3 (3_BH): 8.42 ng (Figure 5C); patient 16 (16_BH): 5.69 ng (Figure 5D); patient 19 (19_BH): 10.96 ng (Figure 5D); patient 25 (25_BH): 14.72 ng (Figure 5E); and patient 26 (26_BH): 18.62 ng (Figure 5E). Then, we have performed serial dilutions of these BHs that were subjected to PMCA analysis together with the corresponding OM samples. We have observed that after 3 rounds, prions were detected in the OM of patient 3 (3_OM). The last PrP^res^ dilution amplified from BH at the 3^rd^ round of this patient corresponded to 8.4 × 10^–11^ g, thus, by postulating that prions contained in BH and OM possess the same seeding activity by PMCA, this might be the approximate concentration of PrP^res^ present in 0.8 μg of OM (Figure 5C). Considering the rapidity of PrP^res^ amplification in this sample, we have verified whether it could contain a quantity of prions detectable by Wb even without amplification but we did not see any PK resistant signal (Supplementary Figure 6). The OM of patient 16 (16_OM) showed PrP^res^ at the 5^th^ round while that of patient 19 (19_OM) at the 6^th^ round which, according to the amplification of their brain dilutions, corresponded to 5.69 × 10^–20^g (Figure 5D) and 1.096 × 10^–21^g (Figure 5D) of protein, both detectable in 0.8 μg of sample. Finally, the OM of patient 25 (OM_25) showed PrP^res^ at the 5^th^ round while that of patient 26 (OM_26) at the 3^rd^ round. In this case, according to the last dilutions amplified from the corresponding BHs, we could estimate that PrP^res^ concentrations in 0.8 μg of these samples are approximately 1.47 × 10^–21^g (Figure 5E) and 1.86 × 10^–13^g (Figure 5E), respectively (Supplementary Table 2).

## Discussion

Although the clinical diagnosis of sporadic Creutzfeldt-Jakob disease has been significantly improved during the last decades, the diagnostic confirmation and the identification of individual sCJD subtypes still requires the neuropathological examination of the brain aimed at detecting and characterizing the prion strain. With the development of the seeding aggregation assays (RT-QuIC and PMCA), traces of prions were found in the CSF, urine, blood, skin and olfactory mucosa of patients with different forms of CJD. While the RT-QuIC represents one of the major breakthroughs for the *antemortem* diagnosis of these diseases, it does not provide specific information about the prion strain, thus hampering the possibility to recognize sCJD subtypes and stratify patients when they are alive. This aspect is of fundamental importance since a strain-dependent efficacy of anti-prion compounds has been emerging (Barret et al., 2003; Yung et al., 2004; Cronier et al., 2007; Berry et al., 2013; Ding et al., 2021). In this work, we have evaluated the efficiency of an optimized PMCA to faithfully amplify prion strains across the spectrum of sCJD subtypes using OM samples collected from living patients. We have implemented the protocol published in 2014 (Moda et al., 2014) by using as a reaction substrate the brain homogenates of TgHuMM mice supplemented with two important cofactors: heparin (already known to enhance prion amplification by PMCA) (Yokoyama et al., 2011; Bélondrade et al., 2021) and sodium tripolyphosphate. With these modifications, we could amplify brain-PrP^Sc^ from almost all sCJD subtypes. In general, the amplification was more efficient for prions having at least one valine at codon 129 (MV1, MV2 and VV2) while it was less efficient for prions homozygous for methionine (MM1, MM2T and MM2C), although the substrate contained PrP^C^ with MM at *PRNP*129. Almost all prions that amplified better generated type 1 PrP^res^ with a prevalent di-glycosylated band while the others generated type 2 PrP^res^ with a prevalent di-glycosylated band, with some exceptions. Unfortunately, the VV1 sample did not amplify and could not be classified in one of these categories.

Interestingly, all PrP^res^ amplified from brain samples showed similar sensitivity to proteolytic digestion. They were also less resistant to digestion than their corresponding unamplified strains. Sometimes these differences reached a statistical significance thus supporting the fact that PMCA may not have retained the original sCJD strain features while giving rise to isolates with distinct biochemical properties.

We could not even exclude the possibility that PMCA has selectively amplified classical or atypical prion isolates, underrepresented in some of the sCJD brains analyzed. Although the biochemical analyses performed in this study have highlighted that some sCJD cases showed a co-occurrence of type 1 and type 2 PrP^res^, the lack of immunohistochemical data hampered the possibility to deepen this aspect even further.

Finally, all sCJD prions have been amplified using the same reaction substrate and this might have further contributed to alter the original strain properties. The ability of prions to change properties when challenged either *in vivo* or *in vitro* is not entirely surprising and has already been reported (Brandner and Jaunmuktane, 2017; Rossi et al., 2019; Cassard et al., 2020). For this reason, PMCA results underpin the unpredictable and fascinating behavior of prions but additional studies are needed to better clarify and explain our findings that are neither obvious nor easy to interpret at the time of writing. Certainly, the optimized PMCA represents an optimal tool that can be further improved and finally exploited to study many aspects of sCJD prions (e.g., biology, heterogeneity, replication and adaptation).

As observed for brains, PMCA was able to efficiently amplify prions from the olfactory mucosa of sCJD and gCJD patients, but it did not retain the peculiar strain properties, thus hindering the possibility to recognize prion strains in living patients. In particular, the assay was able to detect prions with 79.3% sensitivity (23/29 OM) and 100% specificity. We have tried to verify whether the OM that did not amplify prions belonged to sCJD patients with particular features. By analyzing the demographic (e.g. sex, age at disease onset, disease duration, time of brushing after disease onset, time of brushing to death), instrumental (e.g. EEG, MRI-DWI) and laboratory data (CSF markers including t-tau, p-tau and 14.3.3) with appropriate statistical tests, we could not find any significant information useful to set these subjects apart from the others. By considering the number of rounds necessary to generate a detectable PrP^res^ in the other positive samples, we have observed that the MM prions amplified earlier than MV or VV. We do not know whether this is due to the fact that MM samples contain more prions than the others or whether they are able to amplify better in PMCA (possibly due to the use of TgHuMM substrate). Notwithstanding, all amplified OM showed type 1 PrP^res^ with an equal representation of the di- and mono-glycosylated bands, except for three MM samples that showed type 2 PrP^res^ with the di-glycosylated band predominant over the others. As already performed for the patients with negative OM, we have analyzed the clinical, instrumental and demographic features of these three patients but we did not identify any possible correlation or explanation useful to decipher their distinct biochemical properties.

We have then tested the PK resistance profiles of the OM amplified products and found that they were very similar between each other (also considering the three MM samples with different PrP^res^ typing and glycoform ratio). Notably, they were all significantly less resistant to PK digestion than the PrP^res^ amplified from the brains. This was observed also in the case of BH and OM samples belonging to the same sCJD patient. Such finding is puzzling since a few indications revealed that the molecular types of PrP^res^ are conserved in the OM collected from autopsied sCJD cases (MM1 and VV2) (Zanusso et al., 2003). Thus, PMCA seems to amplify prions from BH and OM of the same patient which ultimately acquire distinct biochemical properties. It is also surprising that in three patients (11, 22 and 23) we amplified PrP^res^ from the OM but not from the corresponding BHs. One of these patients, the number 23, was recently found to bear an uncommon mutation at position 113 of the *PRNP* which has never been described and whose role in disease onset and progression is still unknown. Finally, we did not amplify PrP^res^ neither from the brain nor from the olfactory mucosa of patient number 13.

Thus, there are still unknown factors that, together with the experimental constraints to which our samples have been subjected during the amplification, have contributed to modulate the efficiency of prion amplification in different biological tissues (brain *vs.* olfactory mucosa) and have influenced their final biochemical properties.

The availability of brain and olfactory mucosa samples collected from the same sCJD patients gave us the unique opportunity to perform quantitative PMCA and observe that the quantity of prions in different OM is remarkably variable. In particular, we have found a statistically significant correlation between the amount of prions in OM and the age at disease onset, regardless of *PRNP*129: younger sCJD patients contained more prions than older subjects (*p* = 0.0003). We have also observed that OM with high amount of prion belonged to patients with high levels of CSF t–tau, but these results were not statistically significant. We have noticed that the only VV2 OM samples that did not amplify belonged to a patient that, compared to all the other VV2 subjects, was not demented at the time of collection. Again, this is just an observation but we believe that at this very moment it is important to accurately describe all findings that might be further verified in future studies. Contrarily, in the case of MV2 and MM1, we have identified OM samples collected from demented patients that did not amplify by PMCA. No other significant correlations between the presence/amount of OM prions and clinical, demographic or laboratory findings were observed.

In conclusion, although the optimized PMCA did not consent to recognize sCJD subtypes from the analysis of OM collected from living patients, it enabled us to estimate for the first time the amount of prions accumulating in this biological tissue. Animal bioassays are currently ongoing to verify the infectious properties of BH, BH_PMCA and OM_PMCA samples once injected in TgHuMM mice and determine whether the newly acquired biochemical features of BH and OM amplified products are also associated with peculiar infectious and neuropathological properties. Finally, it would be interesting to test whether prions can be amplified from other biological samples, including CSF, urine and blood of the same sCJD patient, eventually using the bank vole brains as a reaction substrate that were shown to retain some of the biochemical features of the original strains (Bélondrade et al., 2021). Given the novelty of the study and the lack of scientific information still available in this context, we are not able to clearly explain some of the findings presented in the manuscript. Nevertheless, we hope that in the near future, PMCA will be implemented to the point of enabling detection and recognition of prions using peripheral tissues of sCJD patients, finally leading to a better selection of patients for future clinical trials and eventually consenting to avoid the need for neuropathological confirmatory tests.

## Data Availability Statement

The datasets presented in this study can be found in online repositories. The names of the repository/repositories and accession number(s) can be found in the article/Supplementary Material.

## Ethics Statement

The studies involving human participants were reviewed and approved by Fondazione IRCCS Istituto Neurologico Carlo Besta. The patients/participants provided their written informed consent to participate in this study. The animal study was reviewed and approved by the Italian Ministry of Health (Permit Number 258/2018-PR).

## Author Contributions

FC performed all PMCA analyses and most of the biochemical data, prepared graphs, and analyzed the results. EB and CD performed PMCA analyses of OM samples. EB, CD, and GB performed biochemical analysis. SP and FQ performed OM collection. MR contributed to prepare OM samples for PMCA analysis. MC performed mice and human genotyping. VR, PC, PT, GDF, AG, RE, GD, AE, RC, PP, GZ, FT, and GG selected and characterized patients subjected to OM collection. GL, GS, and LC produced and purified recombinant proteins used for RT-QuIC and qPMCA analyses. GG, PP, AM, and GZ selected brain homogenates for PMCA. GZ, MF, and MB collected and prepared part of the OM samples for PMCA analysis. FM conceived, supervised the work, and wrote the manuscript. All authors reviewed the manuscript.

## Conflict of Interest

The authors declare that the research was conducted in the absence of any commercial or financial relationships that could be construed as a potential conflict of interest.

## Publisher’s Note

All claims expressed in this article are solely those of the authors and do not necessarily represent those of their affiliated organizations, or those of the publisher, the editors and the reviewers. Any product that may be evaluated in this article, or claim that may be made by its manufacturer, is not guaranteed or endorsed by the publisher.

## Abbreviations

RT-QuIC: real-time quaking-induced conversion
PMCA: protein misfolding cyclic amplification
PrP^C^: prion protein
PrP^Sc^: Prion
VPSPr: variably protease-sensitive prionopathy
sCJD: sporadic Creutzfeldt-Jakob disease
sFI: sporadic fatal insomnia
M: methionine
V: valine
PK: Proteinase K
PrP*^res^*: PK-resistant prion
vCJD: variant Creutzfeldt-Jakob disease
rec-PrP: recombinant PrP
CSF: cerebrospinal fluid
OM: olfactory mucosa
AD: Alzheimer’s disease
FTD: frontotemporal dementia
PD: Parkinson’s disease
MSA: multiple system atrophy
PSP: progressive supranuclear palsy
CBD: corticobasal degeneration
MS: multiple sclerosis
BHs: brain homogenates
Wb: Western blot
PBS: phosphate buffer saline
recHuPrP_23–231_: full-length human PrP with methionine at position 129
recHaPrP_90–231_: truncated Syrian hamster PrP
PMSF: phenylmethylsulfonyl fluoride
GdnHCl: guanidine hydrochloride
EDTA: ethylenediaminetetraacetic acid tetrasodium salt
BH_PMCA: amplified products obtained from BH
OM_PMCA: amplified products obtained from OM
PVDF: polyvinylidene difluoride membranes
HRP: horseradish peroxidase
OND: other neurodegenerative/neurological disorders
MMunk: MM samples with unknown PrP*^res^* typing
MVunk: MV samples with unknown PrP*^res^* typing
VVunk: VV samples with unknown PrP*^res^* typing
PNGase F: Peptide-*N*-glycosidase F
EEG: electroencephalogram
MRI: magnetic resonance imaging
MRI-DWI: diffusion-weighted magnetic resonance imaging
*PRNP*: prion protein gene
GPI: glycosylphosphatidylinositol
CNS: central nervous system
LB: luria bertani
IPTG: isopropyl b-D galactopyranoside
SDS-PAGE: sodium dodecyl sulfate-polyacrylamide gel electrophoresis
ThT: thioflavin T
DTT: dithiothreitol.

## References

[B1] Alexander S. K. Rittman T. Xuereb J. H. Bak T. H. Hodges J. R. Rowe J. B. (2014). Validation of the new consensus criteria for the diagnosis of corticobasal degeneration. *J. Neurol. Neurosurg. Psychiatry* 85 925–929. 10.1136/jnnp-2013-307035 24521567 PMC4112495

[B2] Barret A. Tagliavini F. Forloni G. Bate C. Salmona M. Colombo L. (2003). Evaluation of quinacrine treatment for prion diseases. *J. Virol.* 77 8462–8469. 10.1128/jvi.77.15.8462-8469.2003 12857915 PMC165262

[B3] Barria M. A. Lee A. Green A. J. E. Knight R. Head M. W. (2018). Rapid amplification of prions from variant Creutzfeldt-Jakob disease cerebrospinal fluid. *J. Pathol. Clin. Res.* 4 86–92. 10.1002/cjp2.90 29665324 PMC5903693

[B4] Baskakov I. V. (2014). The many shades of prion strain adaptation. *Prion* 8 169–172. 10.4161/pri.27836 24518385 PMC4189885

[B5] Bate C. Nolan W. McHale-Owen H. Williams A. (2016). Sialic acid within the glycosylphosphatidylinositol anchor targets the cellular prion protein to synapses. *J. Biol. Chem.* 291 17093–17101. 10.1074/jbc.M116.731117 27325697 PMC5016113

[B6] Bélondrade M. Nicot S. Mayran C. Bruyere-Ostells L. Almela F. Di Bari M. A. (2021). Sensitive protein misfolding cyclic amplification of sporadic Creutzfeldt-Jakob disease prions is strongly seed and substrate dependent. *Sci. Rep.* 11:4058. 10.1038/s41598-021-83630-1 33603091 PMC7893054

[B7] Berry D. B. Lu D. Geva M. Watts J. C. Bhardwaj S. Oehler A. (2013). Drug resistance confounding prion therapeutics. *Proc. Natl. Acad. Sci. U.S.A.* 110 E4160–E4169. 10.1073/pnas.1317164110 24128760 PMC3816483

[B8] Brandner S. Jaunmuktane Z. (2017). Prion disease: experimental models and reality. *Acta Neuropathol.* 133 197–222. 10.1007/s00401-017-1670-5 28084518 PMC5250673

[B9] Bruce M. E. Will R. G. Ironside J. W. McConnell I. Drummond D. Suttie A. (1997). Transmissions to mice indicate that “new variant” CJD is caused by the BSE agent. *Nature* 389 498–501. 10.1038/39057 9333239

[B10] Cali I. Lavrich J. Moda F. Kofskey D. Nemani S. K. Appleby B. (2019). PMCA-replicated PrPD in urine of vCJD patients maintains infectivity and strain characteristics of brain PrPD: transmission study. *Sci. Rep.* 9:5191. 10.1038/s41598-019-41694-0 30914754 PMC6435672

[B11] Cali I. Puoti G. Smucny J. Curtiss P. M. Cracco L. Kitamoto T. (2020). Co-existence of PrPD types 1 and 2 in sporadic Creutzfeldt-Jakob disease of the VV subgroup: phenotypic and prion protein characteristics. *Sci. Rep.* 10:1503. 10.1038/s41598-020-58446-0 32001774 PMC6992672

[B12] Camacho M. V. Telling G. Kong Q. Gambetti P. Notari S. (2019). Role of prion protein glycosylation in replication of human prions by protein misfolding cyclic amplification. *Lab. Invest.* 99 1741–1748. 10.1038/s41374-019-0282-1 31249376

[B13] Cassard H. Huor A. Espinosa J.-C. Douet J.-Y. Lugan S. Aron N. (2020). Prions from sporadic creutzfeldt-jakob disease patients propagate as strain mixtures. *mBio* 11:e00393-20. 10.1128/mBio.00393-20 32546613 PMC7298703

[B14] Caughey B. (2001). Interactions between prion protein isoforms: the kiss of death? *Trends Biochem. Sci.* 26 235–242. 10.1016/s0968-0004(01)01792-3 11295556

[B15] Chen B. Morales R. Barria M. A. Soto C. (2010). Estimating prion concentration in fluids and tissues by quantitative PMCA. *Nat. Methods* 7 519–520. 10.1038/nmeth.1465 20512142 PMC4049222

[B16] Cochius J. I. Hyman N. Esiri M. M. (1992). Creutzfeldt-Jakob disease in a recipient of human pituitary-derived gonadotrophin: a second case. *J. Neurol. Neurosurg. Psychiatry* 55 1094–1095. 10.1136/jnnp.55.11.1094 1469410 PMC1015303

[B17] Collinge J. (2010). Prion strain mutation and selection. *Science* 328 1111–1112. 10.1126/science.1190815 20508117

[B18] Concha-Marambio L. Pritzkow S. Moda F. Tagliavini F. Ironside J. W. Schulz P. E. (2016). Detection of prions in blood from patients with variant Creutzfeldt-Jakob disease. *Sci. Transl. Med.* 8:370ra183. 10.1126/scitranslmed.aaf6188 28003548 PMC5538786

[B19] Cronier S. Beringue V. Bellon A. Peyrin J.-M. Laude H. (2007). Prion strain- and species-dependent effects of antiprion molecules in primary neuronal cultures. *J. Virol.* 81 13794–13800. 10.1128/JVI.01502-07 17913812 PMC2168876

[B20] Davidson L. R. R. Llewelyn C. A. Mackenzie J. M. Hewitt P. E. Will R. G. (2014). Variant CJD and blood transfusion: are there additional cases? *Vox Sang.* 107 220–225. 10.1111/vox.12161 24916465

[B21] De Luca C. M. G. Elia A. E. Portaleone S. M. Cazzaniga F. A. Rossi M. Bistaffa E. (2019). Efficient RT-QuIC seeding activity for α-synuclein in olfactory mucosa samples of patients with Parkinson’s disease and multiple system atrophy. *Transl. Neurodegener.* 8:24. 10.1186/s40035-019-0164-x 31406572 PMC6686411

[B22] Ding M. Teruya K. Zhang W. Lee H. W. Yuan J. Oguma A. (2021). Decrease in skin prion-seeding activity of prion-infected mice treated with a compound against human and animal prions: a first possible biomarker for prion therapeutics. *Mol. Neurobiol.* 58 4280–4292. 10.1007/s12035-021-02418-6 33983547 PMC8487418

[B23] Federspil P. A. Federspil P. Plinkert P. K. (2002). Diagnosis of prion diseases. *HNO* 50 327–331. 10.1007/s00106-002-0651-7 12063690

[B24] Franceschini A. Baiardi S. Hughson A. G. McKenzie N. Moda F. Rossi M. (2017). High diagnostic value of second generation CSF RT-QuIC across the wide spectrum of CJD prions. *Sci. Rep.* 7:10655. 10.1038/s41598-017-10922-w 28878311 PMC5587608

[B25] Gambetti P. Dong Z. Yuan J. Xiao X. Zheng M. Alshekhlee A. (2008). A novel human disease with abnormal prion protein sensitive to protease. *Ann. Neurol.* 63 697–708. 10.1002/ana.21420 18571782 PMC2767200

[B26] Gilman S. Wenning G. K. Low P. A. Brooks D. J. Mathias C. J. Trojanowski J. Q. (2008). Second consensus statement on the diagnosis of multiple system atrophy. *Neurology* 71 670–676. 10.1212/01.wnl.0000324625.00404.15 18725592 PMC2676993

[B27] Hamaguchi T. Noguchi-Shinohara M. Nozaki I. Nakamura Y. Sato T. Kitamoto T. (2009). The risk of iatrogenic Creutzfeldt-Jakob disease through medical and surgical procedures. *Neuropathology* 29 625–631. 10.1111/j.1440-1789.2009.01023.x 19659942

[B28] Heath C. A. Barker R. A. Esmonde T. F. G. Harvey P. Roberts R. Trend P. (2006). Dura mater-associated Creutzfeldt-Jakob disease: experience from surveillance in the UK. *J. Neurol. Neurosurg. Psychiatry* 77 880–882. 10.1136/jnnp.2005.073395 16627534 PMC2117491

[B29] Heckmann J. G. Lang C. J. Petruch F. Druschky A. Erb C. Brown P. (1997). Transmission of Creutzfeldt-Jakob disease via a corneal transplant. *J. Neurol. Neurosurg. Psychiatry* 63 388–390. 10.1136/jnnp.63.3.388 9328261 PMC2169722

[B30] Hermann P. Appleby B. Brandel J.-P. Caughey B. Collins S. Geschwind M. D. (2021). Biomarkers and diagnostic guidelines for sporadic Creutzfeldt-Jakob disease. *Lancet Neurol.* 20 235–246. 10.1016/S1474-4422(20)30477-4 33609480 PMC8285036

[B31] Hill A. F. Desbruslais M. Joiner S. Sidle K. C. Gowland I. Collinge J. (1997). The same prion strain causes vCJD and BSE. *Nature* 389 448–450. 10.1038/38925 9333232

[B32] Höglinger G. U. Respondek G. Stamelou M. Kurz C. Josephs K. A. Lang A. E. (2017). Clinical diagnosis of progressive supranuclear palsy: the movement disorder society criteria. *Mov. Disord.* 32 853–864. 10.1002/mds.26987 28467028 PMC5516529

[B33] Hosszu L. L. P. Jackson G. S. Trevitt C. R. Jones S. Batchelor M. Bhelt D. (2004). The residue 129 polymorphism in human prion protein does not confer susceptibility to Creutzfeldt-Jakob disease by altering the structure or global stability of PrPC. *J. Biol. Chem.* 279 28515–28521. 10.1074/jbc.M313762200 15123682

[B34] Ironside J. W. Ritchie D. L. Peden A. H. Head M. W. Kovacs G. G. (2014). “Human prion diseases,” in *Neuropathology of Neurodegenerative Diseases*, Vol. 10 ed. Kovacs G. G. (Cambridge: Cambridge University Press), 176–194. 10.1017/CBO9781107588660.010

[B35] Sanità Istituto Superiore di Istituto Superiore di Sanità Criteri Diagnostici per le Encefalopatie Spongiformi Trasmissibili Umane-Criteri per la Sorveglianza. Available Online at: https://www.iss.it/documents/20126/0/criteri+MCJ_gennaio2017+%281%29.pdf/f7230624-af5b-1c7d-a367-baa728f5bd10?t=1599836838287

[B36] Jones M. Peden A. H. Yull H. Wight D. Bishop M. T. Prowse C. V. (2009). Human platelets as a substrate source for the in vitro amplification of the abnormal prion protein (PrP) associated with variant Creutzfeldt-Jakob disease. *Transfusion* 49 376–384. 10.1111/j.1537-2995.2008.01954.x 18980616

[B37] Kabir M. E. Safar J. G. (2014). Implications of prion adaptation and evolution paradigm for human neurodegenerative diseases. *Prion* 8 111–116. 10.4161/pri.27661 24401672 PMC7030914

[B38] Kobayashi A. Iwasaki Y. Otsuka H. Yamada M. Yoshida M. Matsuura Y. (2013). Deciphering the pathogenesis of sporadic Creutzfeldt-Jakob disease with codon 129 M/V and type 2 abnormal prion protein. *Acta Neuropathol. Commun.* 1:74. 10.1186/2051-5960-1-74 24252157 PMC3833290

[B39] Kobayashi A. Teruya K. Matsuura Y. Shirai T. Nakamura Y. Yamada M. (2015). The influence of PRNP polymorphisms on human prion disease susceptibility: an update. *Acta Neuropathol.* 130 159–170. 10.1007/s00401-015-1447-7 26022925

[B40] Kübler E. Oesch B. Raeber A. J. (2003). Diagnosis of prion diseases. *Br. Med. Bull.* 66 267–279. 10.1093/bmb/66.1.267 14522864

[B41] Ladogana A. Puopolo M. Croes E. A. Budka H. Jarius C. Collins S. (2005). Mortality from Creutzfeldt-Jakob disease and related disorders in Europe, Australia, and Canada. *Neurology* 64 1586–1591. 10.1212/01.WNL.0000160117.56690.B2 15883321

[B42] Li J. Browning S. Mahal S. P. Oelschlegel A. M. Weissmann C. (2010). Darwinian evolution of prions in cell culture. *Science* 327 869–872. 10.1126/science.1183218 20044542 PMC2848070

[B43] Makarava N. Baskakov I. V. (2012). Genesis of tramsmissible protein states via deformed templating. *Prion* 6 252–255. 10.4161/pri.19930 22561163 PMC3399541

[B44] Makarava N. Baskakov I. V. (2013). The evolution of transmissible prions: the role of deformed templating. *PLoS Pathog.* 9:e1003759. 10.1371/journal.ppat.1003759 24339773 PMC3855475

[B45] Mammana A. Baiardi S. Rossi M. Franceschini A. Donadio V. Capellari S. (2020). Detection of prions in skin punch biopsies of Creutzfeldt-Jakob disease patients. *Ann. Clin. Transl. Neurol.* 7 559–564. 10.1002/acn3.51000 32141717 PMC7187701

[B46] McKhann G. M. Knopman D. S. Chertkow H. Hyman B. T. Jack C. R. Kawas C. H. (2011). The diagnosis of dementia due to Alzheimer’s disease: recommendations from the National Institute on Aging-Alzheimer’s Association workgroups on diagnostic guidelines for Alzheimer’s disease. *Alzheimers Dement.* 7 263–269. 10.1016/j.jalz.2011.03.005 21514250 PMC3312024

[B47] Meyer R. K. McKinley M. P. Bowman K. A. Braunfeld M. B. Barry R. A. Prusiner S. B. (1986). Separation and properties of cellular and scrapie prion proteins. *Proc. Natl. Acad. Sci. U.S.A.* 83 2310–2314. 10.1073/pnas.83.8.2310 3085093 PMC323286

[B48] Moda F. Gambetti P. Notari S. Concha-Marambio L. Catania M. Park K.-W. (2014). Prions in the urine of patients with variant Creutzfeldt-Jakob disease. *N. Engl. J. Med.* 371 530–539. 10.1056/NEJMoa1404401 25099577 PMC4162740

[B49] Morales R. Hu P. P. Duran-Aniotz C. Moda F. Diaz-Espinoza R. Chen B. (2016). Strain-dependent profile of misfolded prion protein aggregates. *Sci. Rep.* 6:20526. 10.1038/srep20526 26877167 PMC4753423

[B50] Notari S. Capellari S. Langeveld J. Giese A. Strammiello R. Gambetti P. (2007). A refined method for molecular typing reveals that co-occurrence of PrP(Sc) types in Creutzfeldt-Jakob disease is not the rule. *Lab. Invest.* 87 1103–1112. 10.1038/labinvest.3700676 17893675

[B51] Oesch B. Westaway D. Wälchli M. McKinley M. P. Kent S. B. H. Aebersold R. (1985). A cellular gene encodes scrapie PrP 27-30 protein. *Cell* 40 735–746. 10.1016/0092-8674(85)90333-2 2859120

[B52] Orrú C. D. Bongianni M. Tonoli G. Ferrari S. Hughson A. G. Groveman B. R. (2014). A test for Creutzfeldt-Jakob disease using nasal brushings. *N. Engl. J. Med.* 371 519–529. 10.1056/NEJMoa1315200 25099576 PMC4186748

[B53] Orrú C. D. Yuan J. Appleby B. S. Li B. Li Y. Winner D. (2017). Prion seeding activity and infectivity in skin samples from patients with sporadic Creutzfeldt-Jakob disease. *Sci. Transl. Med.* 9:eaam7785. 10.1126/scitranslmed.aam7785 29167394 PMC5744860

[B54] Pan K. M. Baldwin M. Nguyen J. Gasset M. Serban A. Groth D. (1993). Conversion of alpha-helices into beta-sheets features in the formation of the scrapie prion proteins. *Proc. Natl. Acad. Sci. U.S.A.* 90 10962–10966. 10.1073/pnas.90.23.10962 7902575 PMC47901

[B55] Parchi P. Castellani R. Capellari S. Ghetti B. Young K. Chen S. G. (1996). Molecular basis of phenotypic variability in sporadic Creutzfeldt-Jakob disease. *Ann. Neurol.* 39 767–778. 10.1002/ana.410390613 8651649

[B56] Parchi P. de Boni L. Saverioni D. Cohen M. L. Ferrer I. Gambetti P. (2012). Consensus classification of human prion disease histotypes allows reliable identification of molecular subtypes: an inter-rater study among surveillance centres in Europe and USA. *Acta Neuropathol.* 124 517–529. 10.1007/s00401-012-1002-8 22744790 PMC3725314

[B57] Parchi P. Giese A. Capellari S. Brown P. Schulz-Schaeffer W. Windl O. (1999). Classification of sporadic Creutzfeldt-Jakob disease based on molecular and phenotypic analysis of 300 subjects. *Ann. Neurol.* 46 224–233. 10443888

[B58] Parchi P. Strammiello R. Notari S. Giese A. Langeveld J. P. M. Ladogana A. (2009). Incidence and spectrum of sporadic Creutzfeldt-Jakob disease variants with mixed phenotype and co-occurrence of PrPSc types: an updated classification. *Acta Neuropathol.* 118 659–671. 10.1007/s00401-009-0585-1 19718500 PMC2773124

[B59] Polymenidou M. Stoeck K. Glatzel M. Vey M. Bellon A. Aguzzi A. (2005). Coexistence of multiple PrPSc types in individuals with Creutzfeldt-Jakob disease. *Lancet Neurol.* 4 805–814. 10.1016/S1474-4422(05)70225-8 16297838

[B60] Postuma R. B. Berg D. Stern M. Poewe W. Olanow C. W. Oertel W. (2015). MDS clinical diagnostic criteria for Parkinson’s disease. *Mov. Disord.* 30 1591–1601. 10.1002/mds.26424 26474316

[B61] Prusiner S. B. (1982). Novel proteinaceous infectious particles cause scrapie. *Science* 216 136–144. 10.1126/science.6801762 6801762

[B62] Puoti G. Bizzi A. Forloni G. Safar J. G. Tagliavini F. Gambetti P. (2012). Sporadic human prion diseases: molecular insights and diagnosis. *Lancet Neurol.* 11 618–628. 10.1016/S1474-4422(12)70063-7 22710755

[B63] Puoti G. Giaccone G. Rossi G. Canciani B. Bugiani O. Tagliavini F. (1999). Sporadic Creutzfeldt-Jakob disease: co-occurrence of different types of PrP(Sc) in the same brain. *Neurology* 53 2173–2176. 10.1212/wnl.53.9.2173 10599800

[B64] Rascovsky K. Hodges J. R. Knopman D. Mendez M. F. Kramer J. H. Neuhaus J. (2011). Sensitivity of revised diagnostic criteria for the behavioural variant of frontotemporal dementia. *Brain* 134 2456–2477. 10.1093/brain/awr179 21810890 PMC3170532

[B65] Raymond G. J. Race B. Orrú C. D. Raymond L. D. Bongianni M. Fiorini M. (2020). Transmission of CJD from nasal brushings but not spinal fluid or RT-QuIC product. *Ann. Clin. Transl. Neurol.* 7 932–944. 10.1002/acn3.51057 32538552 PMC7318090

[B66] Redaelli V. Bistaffa E. Zanusso G. Salzano G. Sacchetto L. Rossi M. (2017). Detection of prion seeding activity in the olfactory mucosa of patients with Fatal Familial Insomnia. *Sci. Rep.* 7:46269. 10.1038/srep46269 28387370 PMC5384244

[B67] Ritchie D. L. Ironside J. W. (2017). Neuropathology of human prion diseases. *Prog. Mol. Biol. Transl. Sci.* 150 319–339. 10.1016/bs.pmbts.2017.06.011 28838666

[B68] Rossi M. Baiardi S. Parchi P. (2019). Understanding prion strains: evidence from studies of the disease forms affecting humans. *Viruses* 11:309. 10.3390/v11040309 30934971 PMC6520670

[B69] Rudd P. M. Endo T. Colominas C. Groth D. Wheeler S. F. Harvey D. J. (1999). Glycosylation differences between the normal and pathogenic prion protein isoforms. *Proc. Natl. Acad. Sci. U.S.A.* 96 13044–13049. 10.1073/pnas.96.23.13044 10557270 PMC23897

[B70] Safar J. G. (2012). Molecular pathogenesis of sporadic prion diseases in man. *Prion* 6 108–115. 10.4161/pri.18666 22421210 PMC3366352

[B71] Stahl N. Borchelt D. R. Hsiao K. Prusiner S. B. (1987). Scrapie prion protein contains a phosphatidylinositol glycolipid. *Cell* 51 229–240. 10.1016/0092-8674(87)90150-4 2444340

[B72] Tahiri-Alaoui A. Gill A. C. Disterer P. James W. (2004). Methionine 129 variant of human prion protein oligomerizes more rapidly than the valine 129 variant: implications for disease susceptibility to Creutzfeldt-Jakob disease. *J. Biol. Chem.* 279 31390–31397. 10.1074/jbc.M401754200 15131108

[B73] Telling G. C. Scott M. Hsiao K. K. Foster D. Yang S. L. Torchia M. (1994). Transmission of Creutzfeldt-Jakob disease from humans to transgenic mice expressing chimeric human-mouse prion protein. *Proc. Natl. Acad. Sci. U.S.A.* 91 9936–9940. 10.1073/pnas.91.21.9936 7937921 PMC44932

[B74] Uro-Coste E. Cassard H. Simon S. Lugan S. Bilheude J.-M. Perret-Liaudet A. (2008). Beyond PrP res type 1/type 2 dichotomy in Creutzfeldt-Jakob disease. *PLoS Pathog.* 4:e1000029. 10.1371/journal.ppat.1000029 18383623

[B75] Watson N. Hermann P. Ladogana A. Denouel A. Baiardi S. Colaizzo E. (2022). Validation of revised international creutzfeldt-jakob disease surveillance network diagnostic criteria for sporadic Creutzfeldt-Jakob disease. *JAMA Netw. Open* 5:e2146319. 10.1001/jamanetworkopen.2021.46319 35099544 PMC8804913

[B76] World Health Organisation [WHO] (2003). WHO Manual for Surveillance of Human Transmissible Spongiform Encephalopathies Including Variant Creutzfeldt-Jakob Disease. [Accessed Online at: https://apps.who.int/iris/handle/10665/42656] (accessed February 23, 2022).

[B77] Yokoyama T. Takeuchi A. Yamamoto M. Kitamoto T. Ironside J. W. Morita M. (2011). Heparin enhances the cell-protein misfolding cyclic amplification efficiency of variant Creutzfeldt-Jakob disease. *Neurosci. Lett.* 498 119–123. 10.1016/j.neulet.2011.04.072 21565253

[B78] Yung L. Huang Y. Lessard P. Legname G. Lin E. T. Baldwin M. (2004). Pharmacokinetics of quinacrine in the treatment of prion disease. *BMC Infect. Dis.* 4:53. 10.1186/1471-2334-4-53 15569390 PMC535929

[B79] Zanusso G. Ferrari S. Cardone F. Zampieri P. Gelati M. Fiorini M. (2003). Detection of pathologic prion protein in the olfactory epithelium in sporadic Creutzfeldt-Jakob disease. *N. Engl. J. Med.* 348 711–719. 10.1056/NEJMoa022043 12594315

[B80] Zerr I. Parchi P. (2018). Sporadic Creutzfeldt-Jakob disease. *Handb. Clin. Neurol.* 153 155–174. 10.1016/B978-0-444-63945-5.00009-X 29887134

