# Corrigendum: PMCA-based detection of prions in the olfactory mucosa of patients with sporadic Creutzfeldt–Jakob disease

**DOI:** 10.3389/fnagi.2023.1073356

**Published:** 2023-02-27

**Authors:** Federico Angelo Cazzaniga, Edoardo Bistaffa, Chiara Maria Giulia De Luca, Sara Maria Portaleone, Marcella Catania, Veronica Redaelli, Irene Tramacere, Giuseppe Bufano, Martina Rossi, Paola Caroppo, Anna Rita Giovagnoli, Pietro Tiraboschi, Giuseppe Di Fede, Roberto Eleopra, Grazia Devigili, Antonio Emanuele Elia, Roberto Cilia, Michele Fiorini, Matilde Bongianni, Giulia Salzano, Luigi Celauro, Federico Giuseppe Quarta, Angela Mammana, Giuseppe Legname, Fabrizio Tagliavini, Piero Parchi, Gianluigi Zanusso, Giorgio Giaccone, Fabio Moda

**Affiliations:** ^1^Unit of Neurology 5 and Neuropathology, Fondazione IRCCS Istituto Neurologico Carlo Besta, Milan, Italy; ^2^Department of Neuroscience, Scuola Internazionale Superiore di Studi Avanzati (SISSA), Trieste, Italy; ^3^Department of Health Sciences, Otolaryngology Unit, ASST Santi Paolo e Carlo Hospital, Università degli Studi di Milano, Milan, Italy; ^4^Department of Research and Clinical Development, Scientific Directorate, Fondazione IRCCS Istituto Neurologico Carlo Besta, Milan, Italy; ^5^Unit of Neurology 1 – Parkinson's and Movement Disorders Unit, Fondazione IRCCS Istituto Neurologico Carlo Besta, Milan, Italy; ^6^Department of Neurosciences, Biomedicine and Movement Sciences, University of Verona, Verona, Italy; ^7^IRCCS, Istituto delle Scienze Neurologiche di Bologna (ISNB), Bologna, Italy; ^8^Scientific Directorate, Fondazione IRCCS Istituto Neurologico Carlo Besta, Milan, Italy; ^9^Department of Diagnostic Experimental and Specialty Medicine (DIMES), University of Bologna, Bologna, Italy

**Keywords:** Creutzfeldt–Jakob disease, olfactory mucosa, protein misfolding cyclic amplification, neurodegeneration, prion, peripheral biomarker

In the published article, there was an error in Figure 5 as published. In particular, the Western blot showed in the original Figure 5D related to the 1^st^ round of PMCA of patient 19 (19_BH) was mistakenly selected and has now been replaced with the correct one. The corrected Figure 5D and its caption appear below.

**Figure 5:**
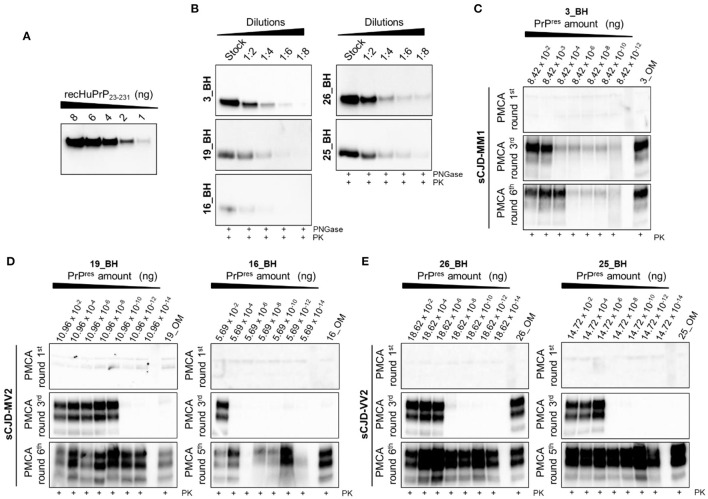
Quantitative PMCA (qPMCA) for estimating PrP^res^ concentration in OM samples of sCJD patients. **(A)** Serial dilutions of recombinant full-length human PrP (recHuPrP_23 − 231_) were used to estimate prion concentration in the brain of sCJD patients. **(B)** Serial dilutions of sCJD brain homogenates subjected to PK and PNGase treatments before Wb analysis. Quantitative PMCA to estimate PrP^res^ concentration in OM of **(C)** MM1, **(D)** MV2, and **(E)** VV2 patients. Specific rounds at which every OM PrP^res^ was detected (3^rd^ for the MM1 and one VV2, 5^th^ for one MV2 and one VV2, and 6^th^ for one MV2) are shown.

The authors apologize for this error and state that this does not change the scientific conclusions of the article in any way. The original article has been updated.

