# Supplementary material for: PMCA-Based Detection of Prions in the Olfactory Mucosa of Patients With Sporadic Creutzfeldt–Jakob Disease
Source: Front Aging Neurosci. 2022 Mar 25;14:848991. doi: 10.3389/fnagi.2022.848991 (PMC8990253; doi:10.3389/fnagi.2022.848991)
Supplement: Supplementary file 1 [file Data_Sheet_1.pdf]

Supplementary material

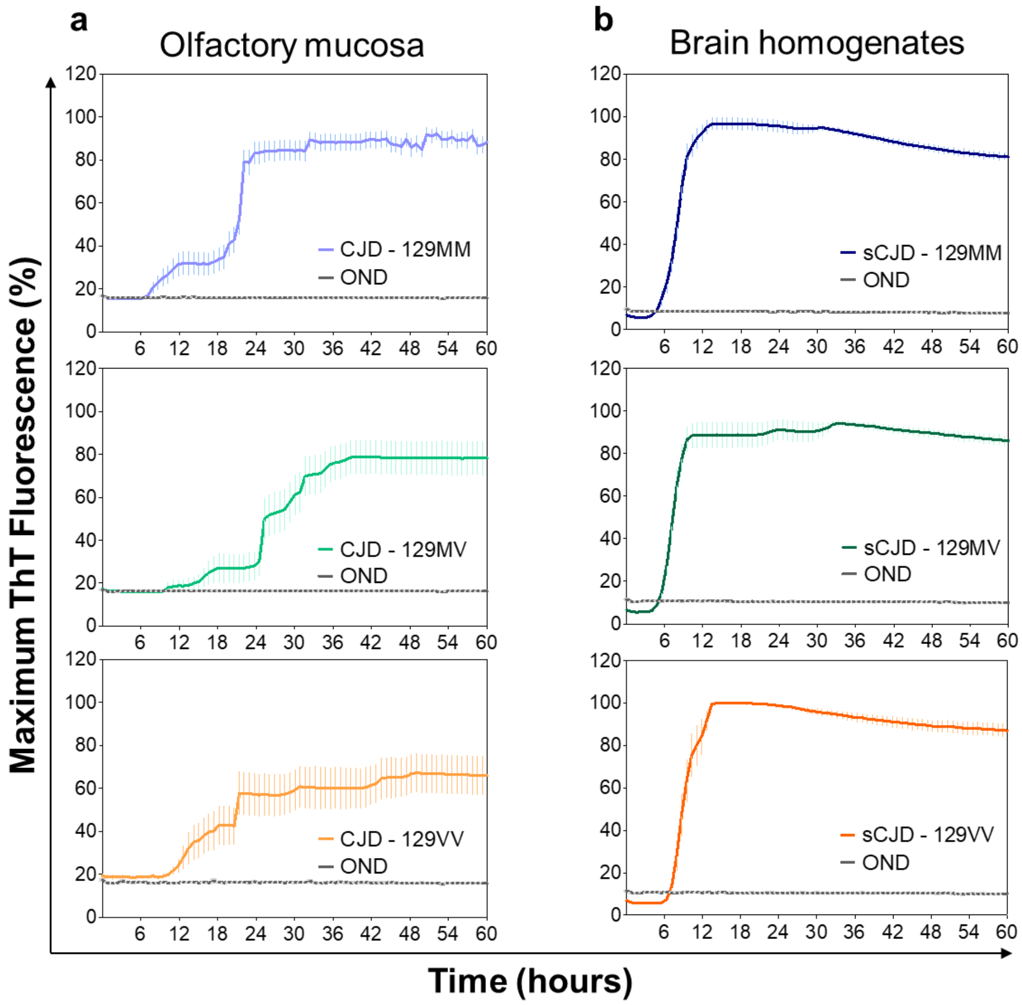

Supplementary Fig. 1 RT-QuIC results of the OM and BHs samples included in the study. (**a**) All OM samples collected from sCJD and gCJD patients (MM=14, light blue line; MV=9, light green line and VV=6, light orange line) induced an efficient RT-QuIC seeding activity, regardless of *PRNP* genotype/mutation, while all OM samples collected from OND (MM=14, MV=16 and VV=6) did not (light grey lines in each graph of group a). (**b**) All BHs collected from sCJD patients (MM=12, dark blue line; MV=9, dark green line; VV=5, dark orange line) induced a rapid RT-QuIC seeding activity while samples collected from OND did not (light grey lines in each graph of group b)

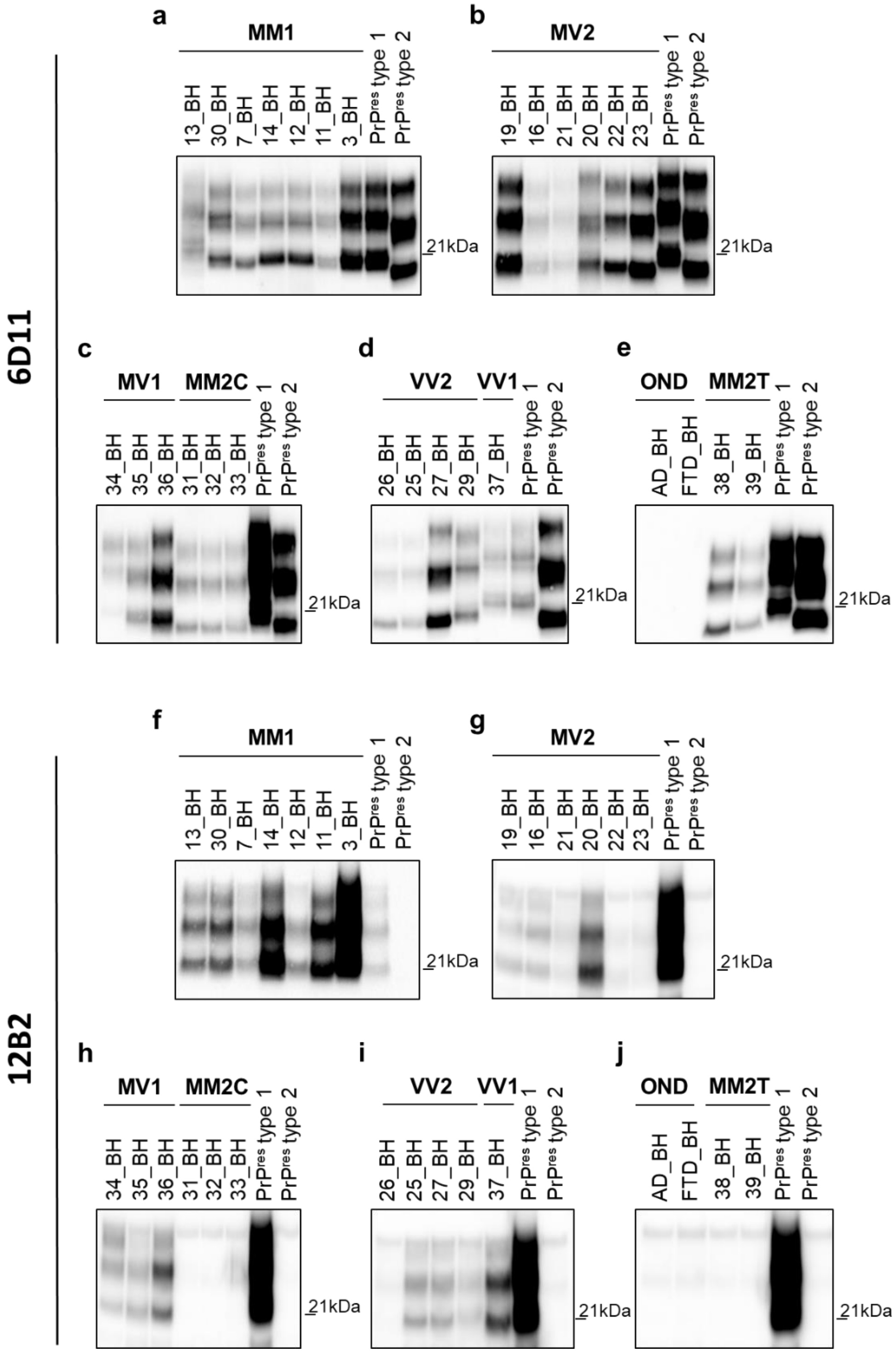

Supplementary Fig. 2 Biochemical analysis of brain homogenates. Western blot (Wb) analysis of BHs enabled to recognize the following sCJD subtypes: (**a-f**) MM1=7, (**b-g**) MV2=6, (**c-h**) MV1=3 and MM2-cortical=3, (**d-i**) VV2=4 and VV1=1, (**e-j**) MM2-thalamic=2 and OND=2. PrP^{res} was detected in all sCJD BHs except for the brains of OND (AD and FTD). The 6D11 monoclonal antibody was used as a probe in a, b, c, d and e, while the 12B2 monoclonal antibody was used as a probe in f, g, h, i and j. The brain homogenates of sCJD patients with known PrP^{res} typing (1 and 2) were used as controls of migration. Numbers in the right of each Wb indicate the molecular weight marker

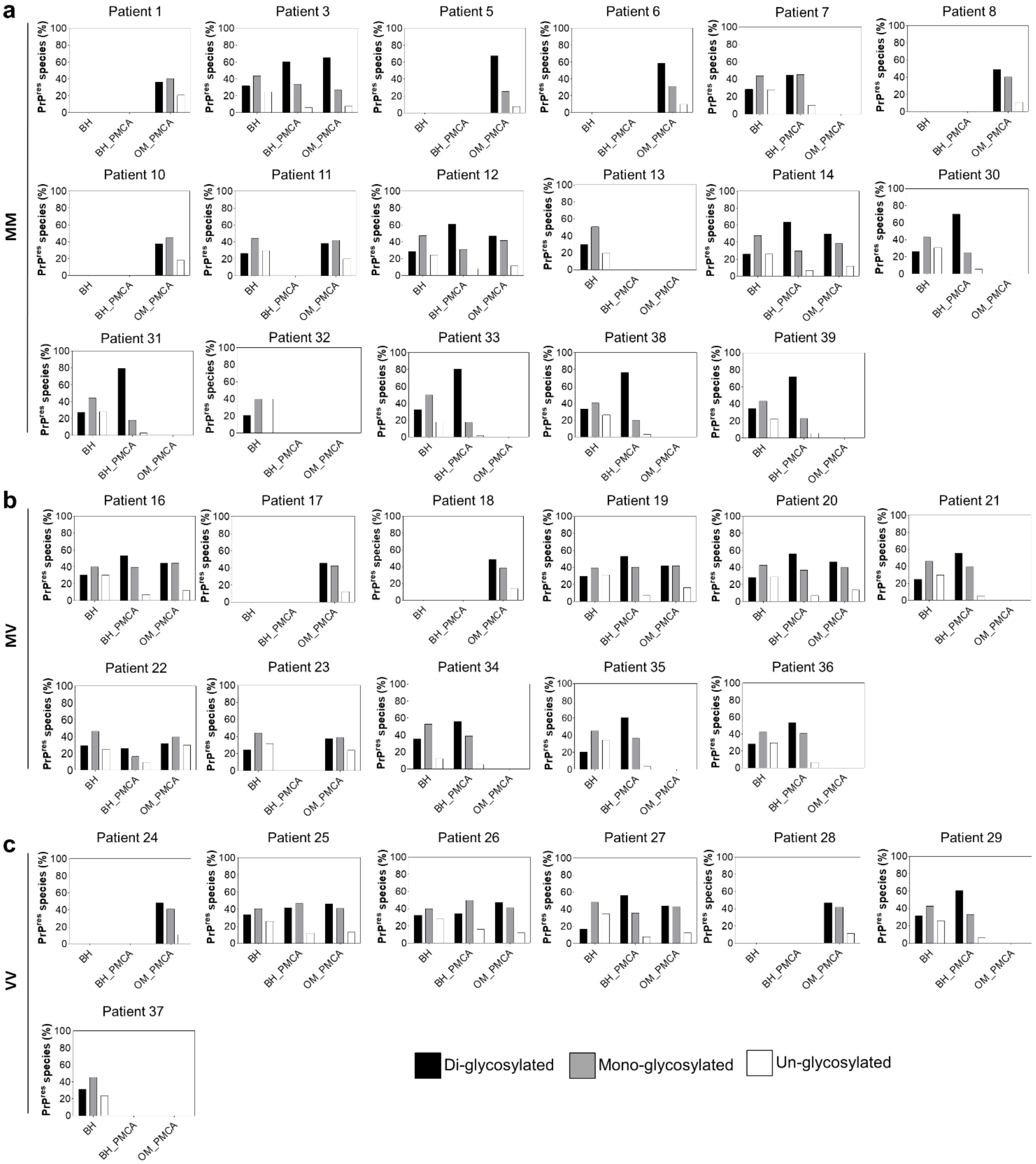

Supplementary Fig. 3 Densitometric analysis of PrP^{res} species of the original strains and their amplification products. Histograms showing the signal intensity of each of PrP^{res} band (di-, mono- and un-glycosylated) from BH and BH_PMCA and/or OM_PMCA of all sCJD patients included in the study (n=39). Samples were divided according to *PRNP*129 (**a**) MM (n=17), (**b**) MV (n=11) and (**c**) VV (n=7). Genetic CJD patients (4 and 15) were not included in the analysis. Two sporadic CJD patients (2 and 9) were also not included since their BHs were not collected and the OM samples remained negative after PMCA

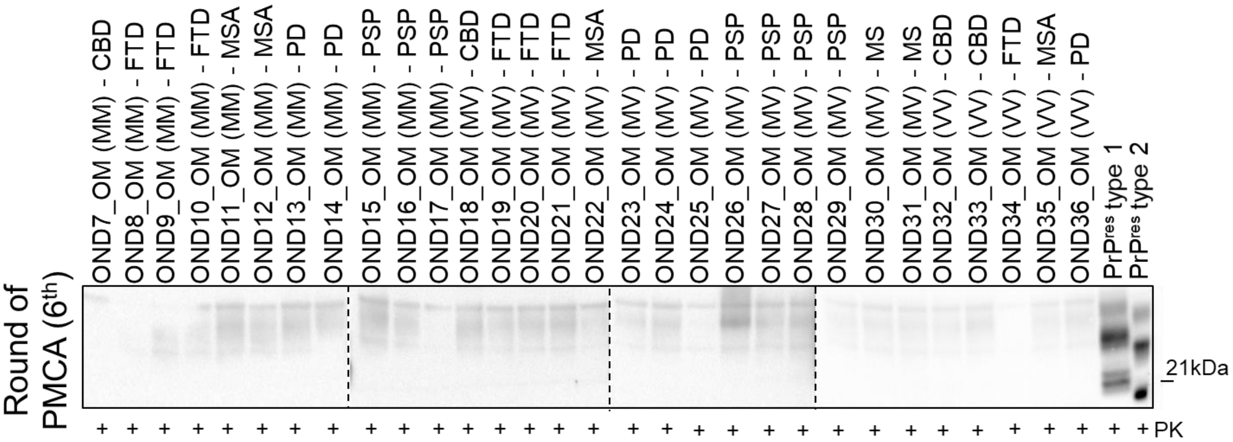

Supplementary Fig. 4 Analysis of olfactory mucosa samples collected from OND patients by PMCA. Wb of the 6th PMCA round is shown. No PrP^{res} was found in the OM of the OND patients with MM (CBD: OND7; FTD: OND8, OND9 and OND10; MSA: OND11 and OND12; PD: OND13 and OND14; PSP: OND15, OND16 and OND17), MV (CBD: OND18; FTD: OND19, OND20 and OND21; MSA: OND22; PD: OND23, OND24 and OND25; PSP: OND26, OND27, OND28 and OND29; MS: OND30 and OND31) and VV (CBD: OND32 and OND33; FTD: OND34; MSA: OND35; PD: OND36) polymorphisms at *PRNP*129. Dashed lines indicate cropped images. Numbers in the right of each Wb indicate the molecular weight marker

		Raw brain homogenates (BHs)		Amplified brain homogenates (BH_PMCA)		Amplified olfactory mucosa samples (OM_PMCA)	
**Patient**	***PRNP***	**Predominant PrP^{res} species**	**PrP^{res} typing**	**Predominant PrP^{res} species**	**PrP^{res} typing**	**Predominant PrP^{res} species**	**PrP^{res} typing**
**3**	MM	Mono	1	Di	2	Di	2
**7**	MM	Mono	1	Di/Mono	1	-	-
**11**	MM	Mono	1	-	-	Di/Mono	1
**12**	MM	Mono	1	Di	2	Di/Mono	1
**13**	MM	Mono	1	-	-	-	-
**14**	MM	Mono	1	Di	2	Di/Mono	1
**16**	MV	Mono	2	Di	1	Di/Mono	1
**19**	MV	Mono	2	Di	1	Di/Mono	1
**20**	MV	Mono	2	Di	1	Di/Mono	1
**21**	MV	Mono	2	Di	1	-	-
**22**	MV	Mono	2	Di	1	Di/Mono	1
**23**	MV	Mono	2	-	-	Di/Mono	1
**25**	VV	Mono	2	Di/Mono	1	Di/Mono	1
**26**	VV	Mono	2	Di/Mono	1	Di/Mono	1
**27**	VV	Mono	2	Di	1	Di/Mono	1
**29**	VV	Mono	2	Di	1	-	-

Table S1 Summary of the biochemical properties of PrP^{res}. The biochemical properties of PrP^{res} present in the brains (BHs) or generated by PMCA from the brains (BH_PMCA) and olfactory mucosa (OM_PMCA) are shown. Di: prevalent di-glycosylated band; Mono: prevalent mono-glycosylated band; Di/Mono: equally representation of di- and mono-glycosylated bands

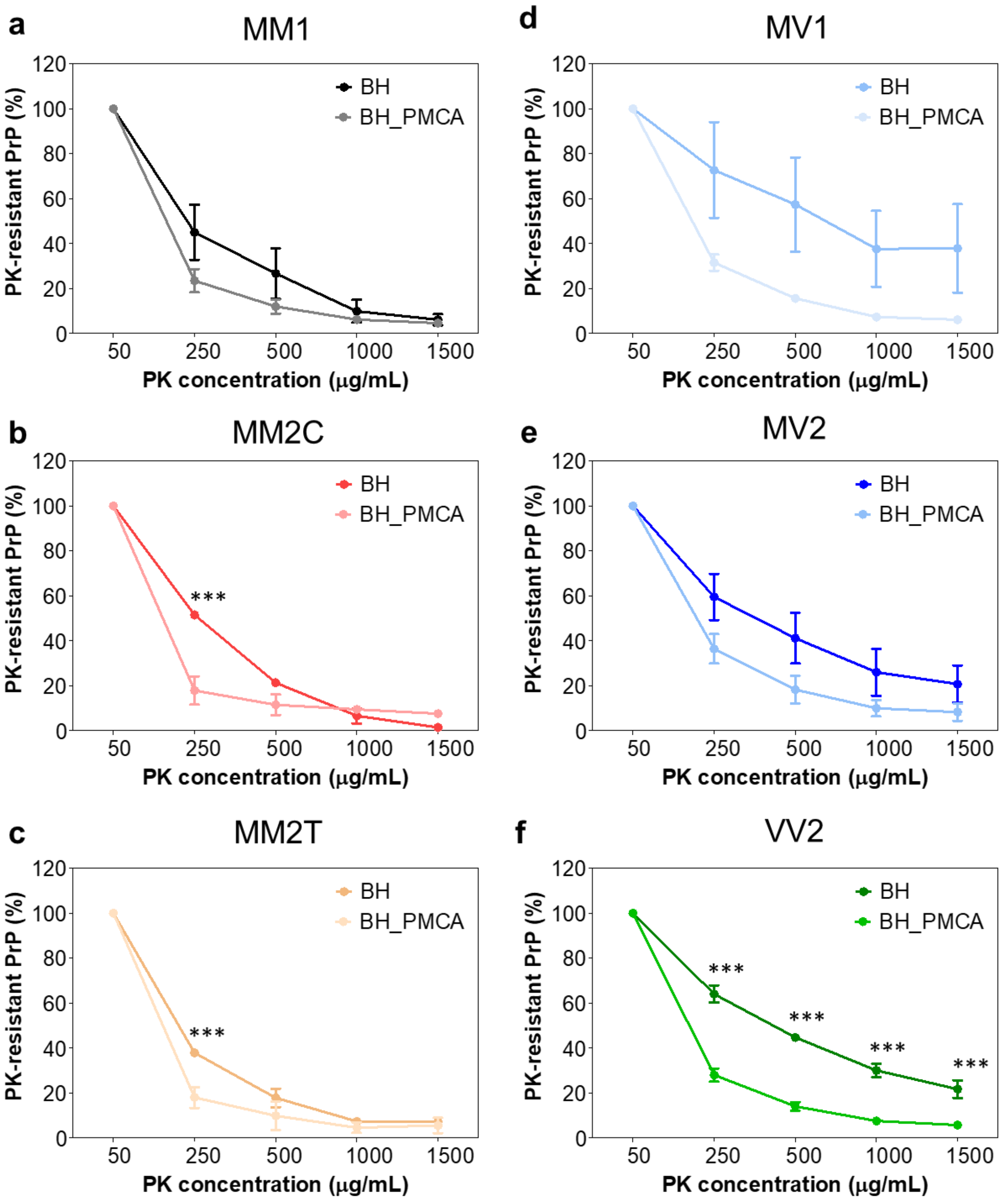

Supplementary Fig. 5 Comparison of PK resistances between BH and their amplified products collected at the 6th PMCA round. PK resistant profiles of BH *vs* BH_PMCA of (**a**) MM1, (**d**) MV1 and (**e**) MV2 patients were different but not in a statistically significant manner. The PK resistant profiles of BH *vs* BH_PMCA of (**b**) MM2C, (**c**) MM2T and (**f**) VV2 showed statistically significant differences. Statistical analyses: repeated measure analysis of variance (ANOVA); BH vs BH_PMCA:***$p<0.001$; error bars: ± standard error of the mean [SEM]

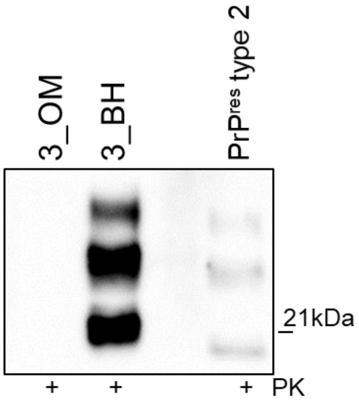

Supplementary Fig. 6 Western blot analysis of OM and BH from a patient with sCJD (MM1). Wb of BH (10% w/v) and OM (2 μg) of patient 3. The 3_OM sample was digested with PK, and no PrP^{res} was found. In contrast, 3_BH sample treated with PK shows a type 1 PrP^{res}. The 6D11 monoclonal antibody was used. The brain homogenate of a sCJD patient with PrP^{res} type 2 was used as a control of migration. Number in the right of Wb indicates the molecular weight marker

	*PRNP*129	BH	OM	Ref. Figure
Patient 3	MM1	8.42 ng	8.42×10^{-11} g	Fig. 5c
Patient 16	MV2	5.69 ng	5.69×10^{-20} g	Fig. 5d
Patient 19	MV2	10.96 ng	1.096×10^{-21} g	Fig. 5d
Patient 25	VV2	14.75 ng	1.475×10^{-21} g	Fig. 5e
Patient 26	VV2	18.62 ng	1.862×10^{-13} g	Fig. 5e

Table S2 Comparison of PrP^{res} concentration in BH and OM samples. Estimation of PrP^{res} concentration in BH and OM samples of MM1 (n=1), MV2 (n=2) and VV2 (n=2) patients obtained by qPMCA analysis.

Cropped images of Western blots

Supplementary Fig. 4 Analysis of olfactory mucosa samples collected from OND patients by PMCA.

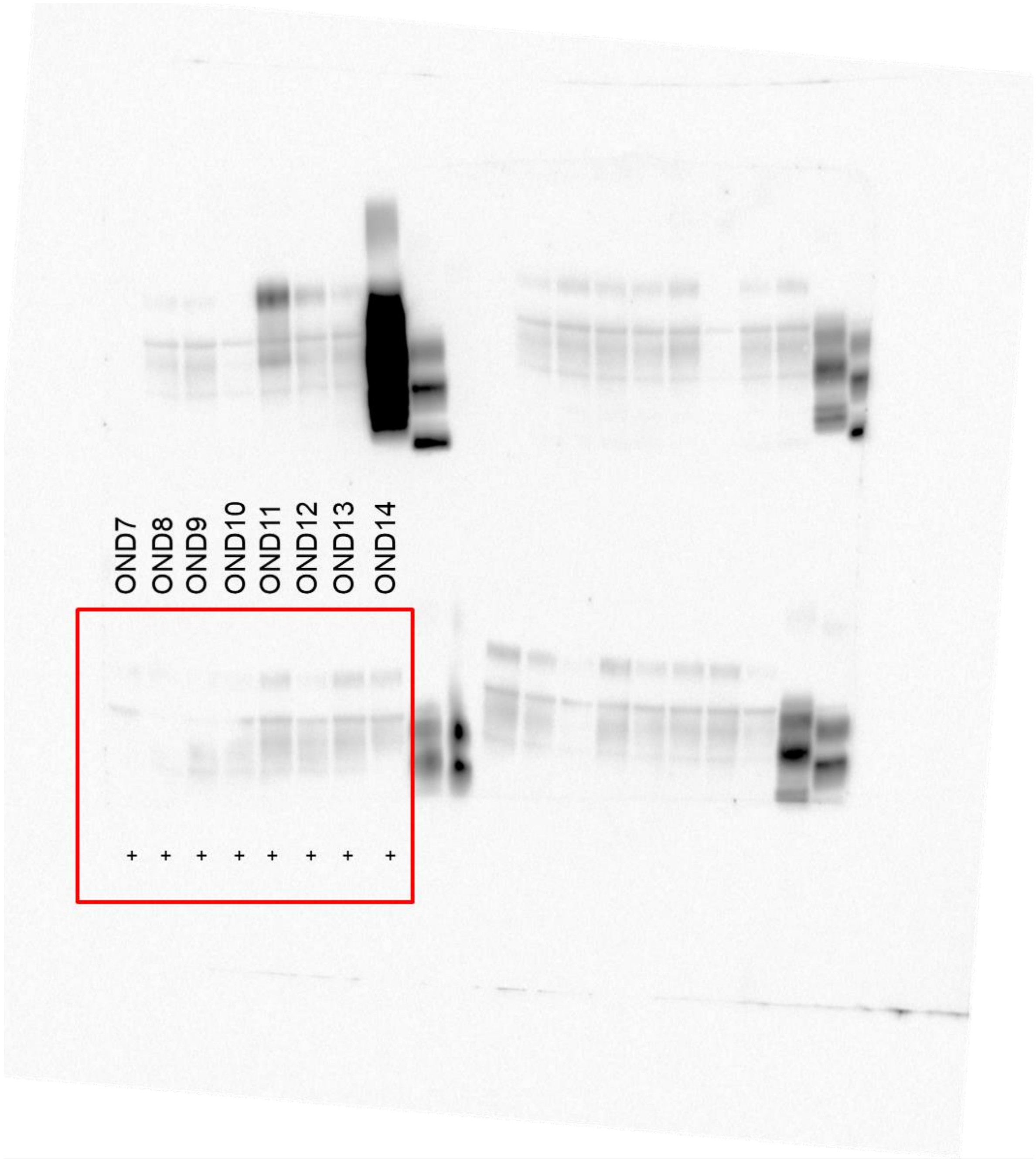

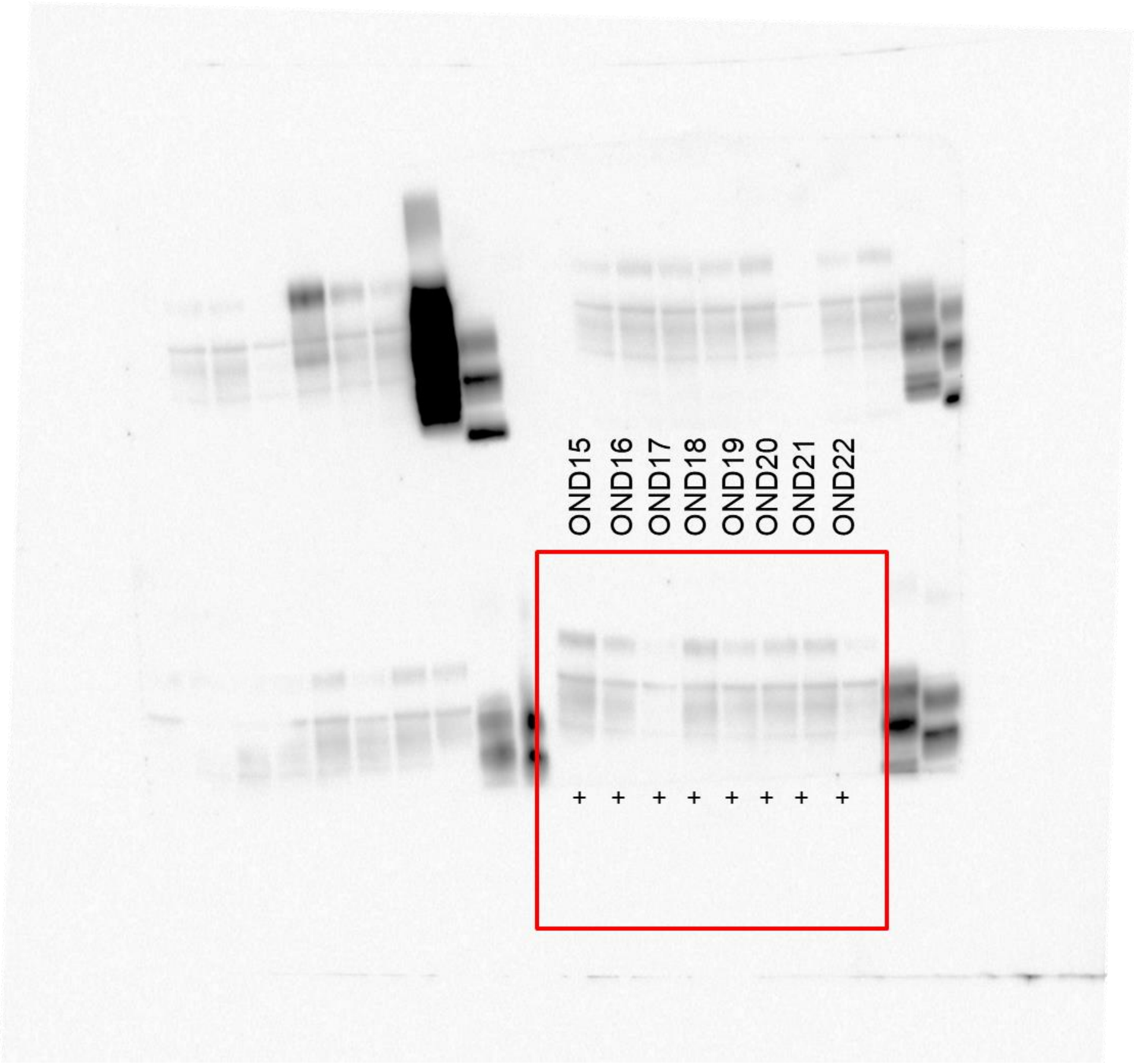
OND15
OND16
OND17
OND18
OND19
OND20
OND21
OND22
+ + + + + + + +

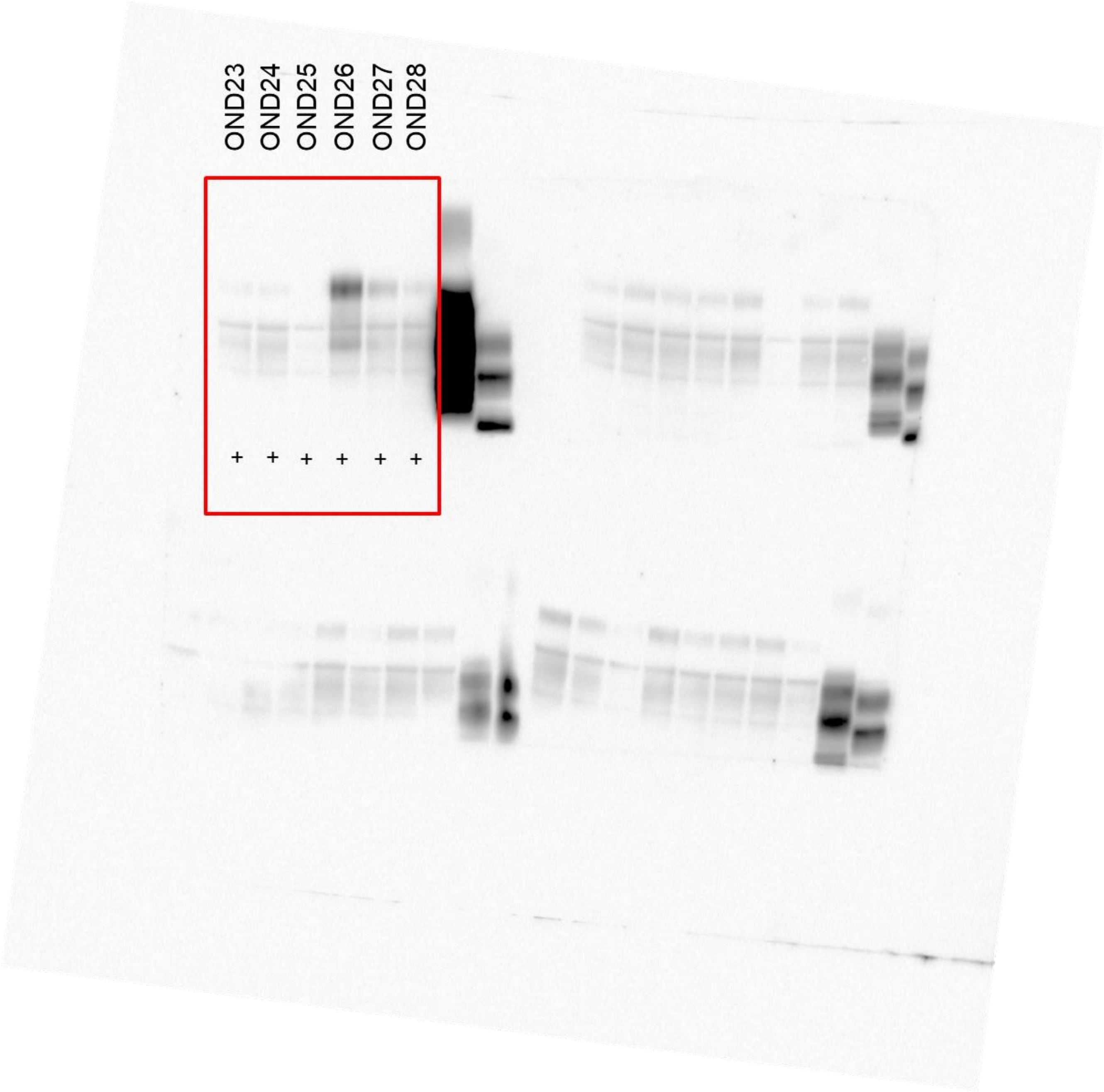
OND23
OND24
OND25
OND26
OND27
OND28

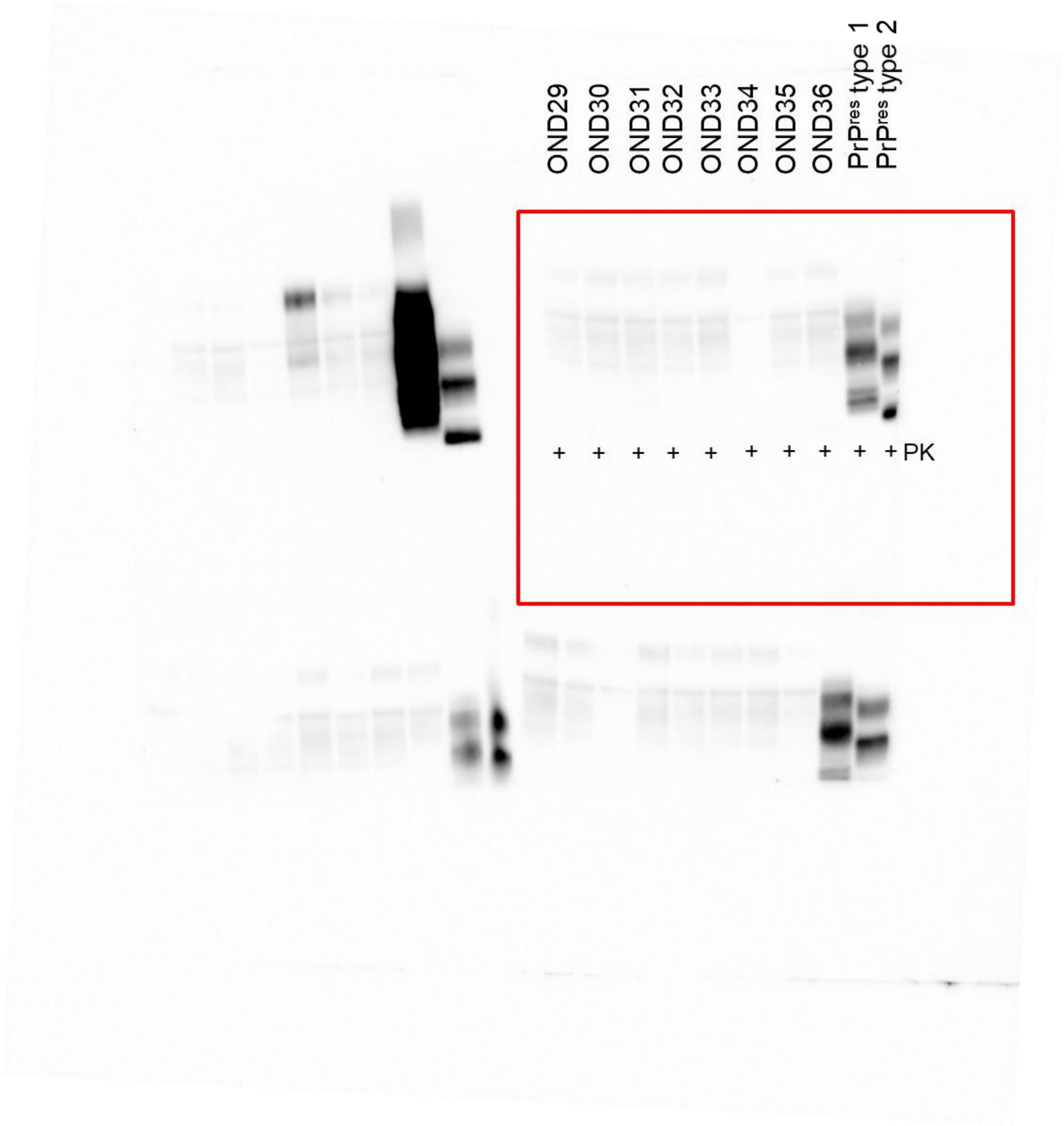
OND29
OND30
OND31
OND32
OND33
OND34
OND35
OND36
PrPres type 1
PrPres type 2
+ + + + + + + + + + PK
